# Carvacrol ameliorates acute campylobacteriosis in a clinical murine infection model

**DOI:** 10.1186/s13099-019-0343-4

**Published:** 2020-01-08

**Authors:** Soraya Mousavi, Anna-Maria Schmidt, Ulrike Escher, Sophie Kittler, Corinna Kehrenberg, Elisa Thunhorst, Stefan Bereswill, Markus M. Heimesaat

**Affiliations:** 1 CC5, Institute of Microbiology, Infectious Diseases and Immunology, Gastrointestinal Microbiology Research Group, Charité - University Medicine Berlin, Corporate Member of Freie Universität Berlin, Humboldt-Universität Zu Berlin, and Berlin Institute of Health, Campus Benjamin Franklin, FEM, Garystr. 5, 14195 Berlin, Germany; 2 Institute for Food Quality and Food Safety, University of Veterinary Medicine Hannover, Hannover, Germany; 3 Institute for Veterinary Food Science, Justus-Liebig-University, Giessen, Germany

**Keywords:** Carvacrol, Anti-pathogenic and anti-inflammatory properties, *Campylobacter jejuni*, Secondary abiotic IL-10^−/−^ mice, Pro-inflammatory immune responses, Bacterial translocation, Host–pathogen-interaction, Intestinal immunopathology, Extra-intestinal and systemic immune responses

## Abstract

**Background:**

The prevalence of human infections with the zoonotic pathogen *Campylobacter jejuni* is rising worldwide. Therefore, the identification of compounds with potent anti-pathogenic and anti-inflammatory properties for future therapeutic and/or preventive application to combat campylobacteriosis is of importance for global health. Results of recent studies suggested carvacrol (4-isopropyl-2-methylphenol) as potential candidate molecule for the treatment of campylobacteriosis in humans and for the prevention of *Campylobacter* colonization in farm animals.

**Results:**

To address this in a clinical murine infection model of acute campylobacteriosis, secondary abiotic IL-10^−/−^ mice were subjected to synthetic carvacrol via the drinking water starting 4 days before peroral *C. jejuni* challenge. Whereas at day 6 post-infection placebo treated mice suffered from acute enterocolitis, mice from the carvacrol cohort not only harbored two log orders of magnitude lower pathogen loads in their intestines, but also displayed significantly reduced disease symptoms. Alleviated campylobacteriosis following carvacrol application was accompanied by less distinct intestinal apoptosis and pro-inflammatory immune responses as well as by higher numbers of proliferating colonic epithelial cells. Remarkably, the inflammation-ameliorating effects of carvacrol treatment were not restricted to the intestinal tract, but could also be observed in extra-intestinal organs such as liver, kidneys and lungs and, strikingly, systemically as indicated by lower IFN-γ, TNF, MCP-1 and IL-6 serum concentrations in carvacrol versus placebo treated mice. Furthermore, carvacrol treatment was associated with less frequent translocation of viable *C. jejuni* originating from the intestines to extra-intestinal compartments.

**Conclusion:**

The lowered *C. jejuni* loads and alleviated symptoms observed in the here applied clinical murine model for human campylobacteriosis highlight the application of carvacrol as a promising novel option for both, the treatment of campylobacteriosis and hence, for prevention of post-infectious sequelae in humans, and for the reduction of *C. jejuni* colonization in the intestines of vertebrate lifestock animals.

## Background

In the United States, up to 10 million foodborne-related cases were estimated to be responsible for approximately 1300 deaths annually [1]. The presence of distinct bacterial species in livestock farming is associated with food-borne human infections resulting in gastrointestinal and post-infectious extra-intestinal morbidities with rising prevalence rates worldwide [2, 3]. Particularly *Campylobacter* infections are of substantial and increasing importance for food-borne diseases, whereas farm animals, especially poultry, are the main origin of human infection [4–6]. Following ingestion of raw or undercooked contaminated meat or surface water, symptoms of campylobacteriosis may vary considerably [7, 8]. Whereas some patients are even asymptomatic or present with rather mild symptoms, others suffer from abdominal cramps, fever, watery of even bloody and inflammatory diarrhea that usually resolve within 1 week. In rare cases, however, post-infectious sequelae such as Gullain-Barré syndrome, Miller Fisher syndrome, or reactive arthritis may manifest [9–11]. The pathogenesis of acute human campylobacteriosis is strongly triggered by the activation of innate immune responses via Toll-like Receptor-4 (TLR-4) mediated sensing of the bacterial lipooligosaccharide (LOS) that is expressed on the surface of *C. jejuni* [12, 13]. Thus, innate immune responses upon *C. jejuni* infection are very similar to those observed following peracute infections with other LOS expressing pathogens such as *Neisseria meningitidis* and *N. gonorrhoeae* [14, 15].

Terpenoids are antimicrobial compounds that are effective against a broad range of microorganisms [16]. Carvacrol (4-isopropyl-2-methylphenol) is a monoterpenoid which constitutes a major compound in essential oils of thyme and oregano and other medicinal plants with many proven health beneficial effects [17, 18]. Carvacrol modulates a multitude of different enzymatic functions which are causative for its anxiolytic, spasmolytic, cell regenerative and anticancer activities and is further in the focus of infection research due to its natural antimicrobial effects against several food-borne pathogens including *Campylobacter*. In vitro studies revealed that in bacteriostatic concentrations, carvacrol is capable of inducing changes in the fatty acid composition of the bacterial cell walls [19, 20]. In bactericidal concentrations, however, carvacrol even permeabilizes the outer membrane of Gram-negative bacteria [21]. In addition, carvacrol possesses ATPase-inhibiting activity [22, 23] and is proposed to act as a proton exchanger that reduces the pH gradient across the cytoplasmic membrane causing changes in proton motive force and in the ATP pool, which leads to cell death [23, 24]. Both, in vitro and in vivo studies revealed that carvacrol application could effectively reduce *C. jejuni* loads in intestinal samples derived from chicken [25–28]. Furthermore, carvacrol could effectively reduce virulence gene expression and invasion of *C. jejuni* into chicken cells [26, 29]. Most importantly, the finding that carvacrol application resulted in inhibition of motility and invasive properties of *C. jejuni *in vitro points towards carvacrol as a promising candidate molecule for the combat of human campylobacteriosis [30]. Recently, our group has established a clinical murine *C. jejuni* infection model allowing for pre-clinical studies of potential compounds against campylobacteriosis at the pharmaceutical level. After peroral *C. jejuni* infection, secondary abiotic IL-10^−/−^ mice in which the intestinal microbiota had been depleted by antibiotic treatment could be stably colonized with *C. jejuni* at high pathogenic loads [12]. Given the lack of LOS resistance due to the absence of IL-10, these mice display *C. jejuni* induced acute enterocolitis within 1 week post-infection (p.i.) thereby mimicking clinical key features of severe campylobacteriosis [12, 31, 32]. In the present study, we applied this clinical murine model for human campylobacteriosis in order to investigate the therapeutic and/or even preventive efficacies of carvacrol treatment against *C. jejuni* colonization and immunopathological sequelae in vivo.

## Results

### Antimicrobial properties of carvacrol against *C. jejuni* isolates

We first addressed potential directed antimicrobial effects of carvacrol against *C. jejuni*. In vitro studies with 20 *C. jejuni* isolates including the reference strain 81–176 revealed carvacrol MIC_90_ values of 150 mg/l (pH 7.3).

### Gastrointestinal pathogen loads following carvacrol treatment of *C. jejuni* infected mice

**Fig. 1:**
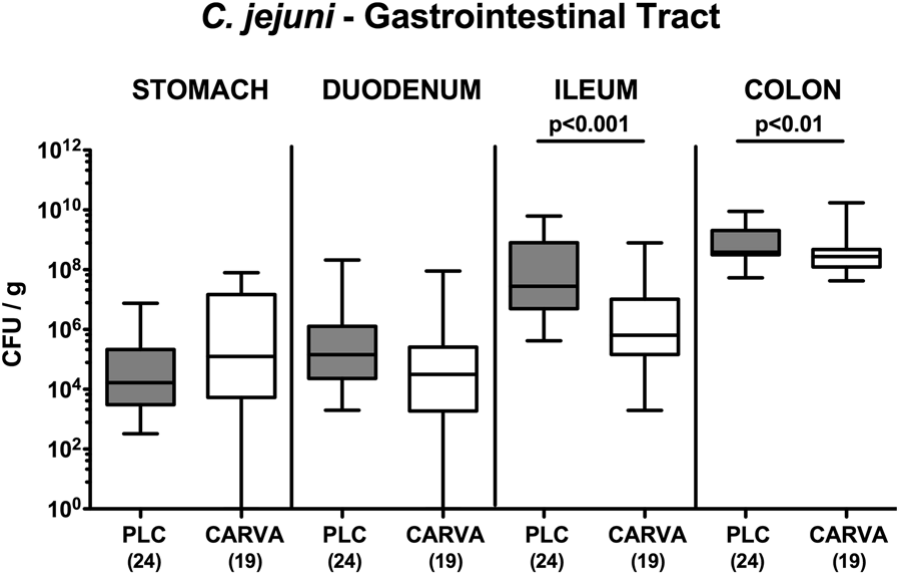
Gastrointestinal pathogen loads following carvacrol treatment of *C. jejuni* infected mice. Starting 4 days prior peroral *C. jejuni* infection on days 0 and 1, secondary abiotic IL-10^−/−^ mice were treated with synthetic carvacrol (CARVA; white boxes) or placebo (PLC; grey boxes) via the drinking water. At day 6 post-infection, *C. jejuni* were cultured from distinct luminal parts of the gastrointestinal tract and pathogen loads expressed as colony forming units per gram (CFU/g). Box plots represent the 75th and 25th percentiles of medians (black bar inside the boxes). The total range, significance levels (*p*-values) determined by the Mann–Whitney U test and numbers of analyzed animals (in parentheses) are indicated. Data were pooled from four independent experiments

We next assessed potential health-beneficial properties (i.e., anti-*C. jejuni* and anti-inflammatory effects) of synthetic carvacrol (500 mg/l via the drinking water, ad libitum) in the here applied clinical murine infection model for the study of severe human campylobacteriosis. To accomplish this, secondary abiotic IL-10^−/−^ mice were treated with carvacrol via the drinking water starting 4 days prior peroral *C. jejuni* infection with 10^9^ bacterial cells by gavage on days 0 and 1. At day 6 p.i., placebo (PLC) treated control mice harbored median pathogen loads of 10^9^ and 10^8^ CFU/g in their colon and ileum, respectively, that were approximately 0.5 and 2.0 log orders of magnitude lower in mice from the carvacrol cohort, respectively (p < 0.01 and p < 0.001, respectively; Fig. 1). Hence, carvacrol treatment lowers intestinal *C. jejuni* burdens up to 2 orders of magnitude.

### Clinical effects upon carvacrol treatment of *C. jejuni* infected mice

**Fig. 2:**
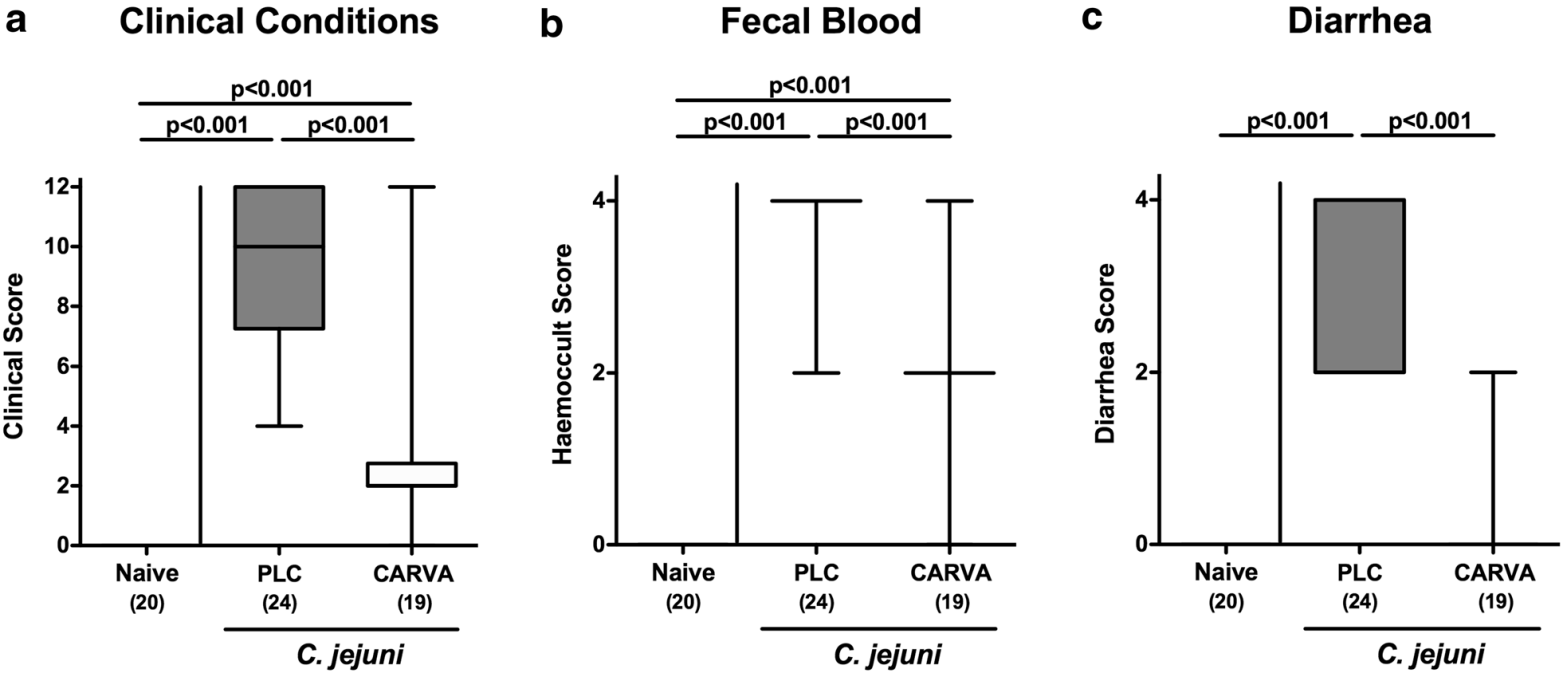
Clinical outcome following carvacrol treatment of *C. jejuni* infected mice. Starting 4 days prior peroral *C. jejuni* infection on days 0 and 1, secondary abiotic IL-10^−/−^ mice were treated with synthetic carvacrol (CARVA; white boxes) or placebo (PLC; grey boxes) via the drinking water. Applying a standardized clinical scoring system (see “Methods”), **a** clinical outcome, **b** abundance of fecal blood and **c** stool consistency were quantitated at day 6 post-infection. Box plots represent the 75th and 25th percentiles of medians (black bar inside the boxes). The total range, significance levels (*p*-values) determined by the Mann–Whitney U test and numbers of analyzed animals (in parentheses) are indicated. Data were pooled from four independent experiments

We further performed a daily survey of *C. jejuni* induced symptoms in infected mice applying a standardized clinical scoring system assessing gross appearance of mice, stool consistency and abundance of blood in fecal samples. As early as 48 h after the latest of two peroral pathogenic challenges (i.e., on day 3 p.i.), mice from the carvacrol group displayed less severe *C. jejuni* induced disease as indicated by lower clinical scores as compared to PLC controls (p < 0.001; Additional file 1: Fig. S1), which also held true for days 5 and 6 p.i. (p < 0.001; Additional file 1: Fig. S1). At necropsy, PLC treated control mice suffered from acute enterocolitis characterized by wasting and bloody diarrhea (Fig. 2), whereas carvacrol treated mice, however, were clinically less compromised as indicated by significantly reduced scores for gross appearance, abundance of fecal blood and diarrhea (p < 0.001 vs. PLC; Fig. 2). Notably, all control mice, but only 10.5% of carvacrol treated animals presented with diarrhea at day 6 p.i. (p < 0.001; Fig. 2c). Hence, carvacrol treatment alleviates *C. jejuni* induced symptoms of campylobacteriosis in the here applied clinical murine infection model.

### Apoptotic and regenerative responses in colonic epithelial cells upon carvacrol treatment of *C. jejuni* infected mice

**Fig. 3:**
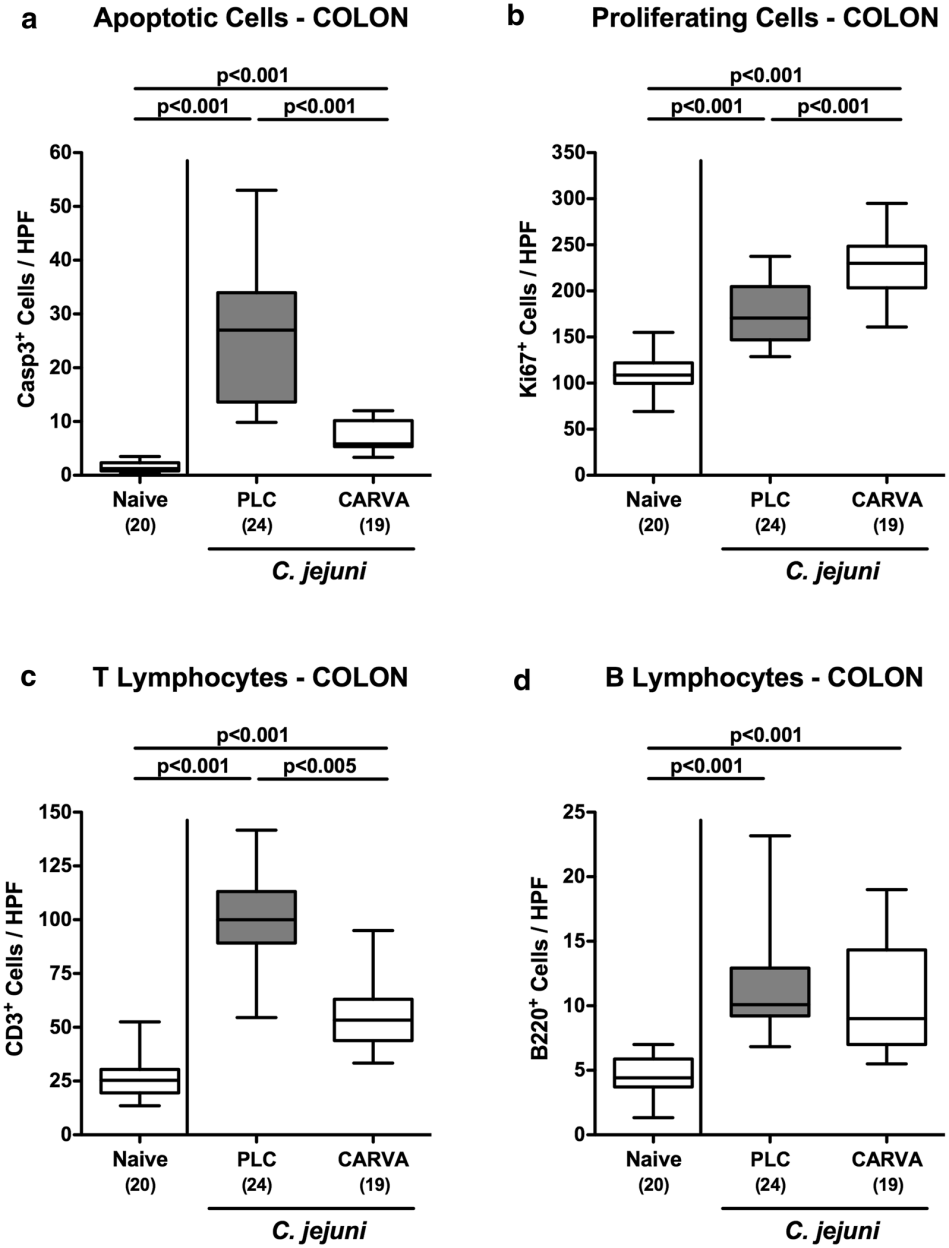
Large intestinal apoptotic, proliferative/regenerative and immune cell responses upon carvacrol treatment of *C. jejuni* infected mice. Starting 4 days prior peroral *C. jejuni* infection on days 0 and 1, secondary abiotic IL-10^−/−^ mice were treated with synthetic carvacrol (CARVA; white boxes) or placebo (PLC; grey boxes) via the drinking water. The average numbers of colonic epithelial **a** apoptotic cells (positive for caspase-3, Casp3) and **b** proliferating/regenerating cells (positive for Ki67) as well as of **c** T lymphocytes (positive for CD3) and **d** B lymphocytes (positive for B220) in the mucosa of lamina propria from six high power fields (HPF, 400x magnification) per mouse were assessed microscopically in immunohistochemically stained large intestinal paraffin sections at day 6 post-infection. Naive mice served as uninfected controls. The total range, significance levels (p-values) determined by the Mann–Whitney U test and numbers of analyzed animals (in parentheses) are indicated. Data were pooled from four independent experiments

We next assessed whether the beneficial effects of carvacrol treatment on the macroscopic outcome of *C. jejuni* infected mice could also be observed on microscopic level. To address this, we stained colonic paraffin sections with defined antibodies against distinct cell inflammatory and proliferative/regenerative markers as well as against distinct immune cell populations applying in situ immunohistochemistry. At day 6 p.i., mice of either cohort exhibited increased numbers of caspase3 positive apoptotic cells in their colonic epithelia (p < 0.001 vs. naive; Fig. 3a). However, numbers of apoptotic cells were five times lower in colonic epithelia of carvacrol as compared to PLC treated mice at day 6 p.i. (p < 0.001; Fig. 3a; Additional file 2: Fig. S2A). We next stained large intestinal paraffin sections with antibodies against Ki67 and quantified the respective cell proliferation and regeneration counteracting *C. jejuni* induced cell damage microscopically. In fact, *C. jejuni* infection was accompanied by a marked increase in Ki67 positive colonic epithelial cells (p < 0.001 vs. naive; Fig. 3b; Additional file 2: Fig. S2B). Importantly, numbers of proliferative/ regenerative cells were significantly elevated in carvacrol as compared to PLC mice at day 6 p.i. (p < 0.001; Fig. 3b; Additional file 2: Fig. S2B). Hence, carvacrol treatment ameliorates murine campylobacteriosis by inhibiting apoptosis and stimulating regenerative processes in the colonic epithelia.

### Colonic immune responses upon carvacrol treatment of *C. jejuni* infected mice

**Fig. 4:**
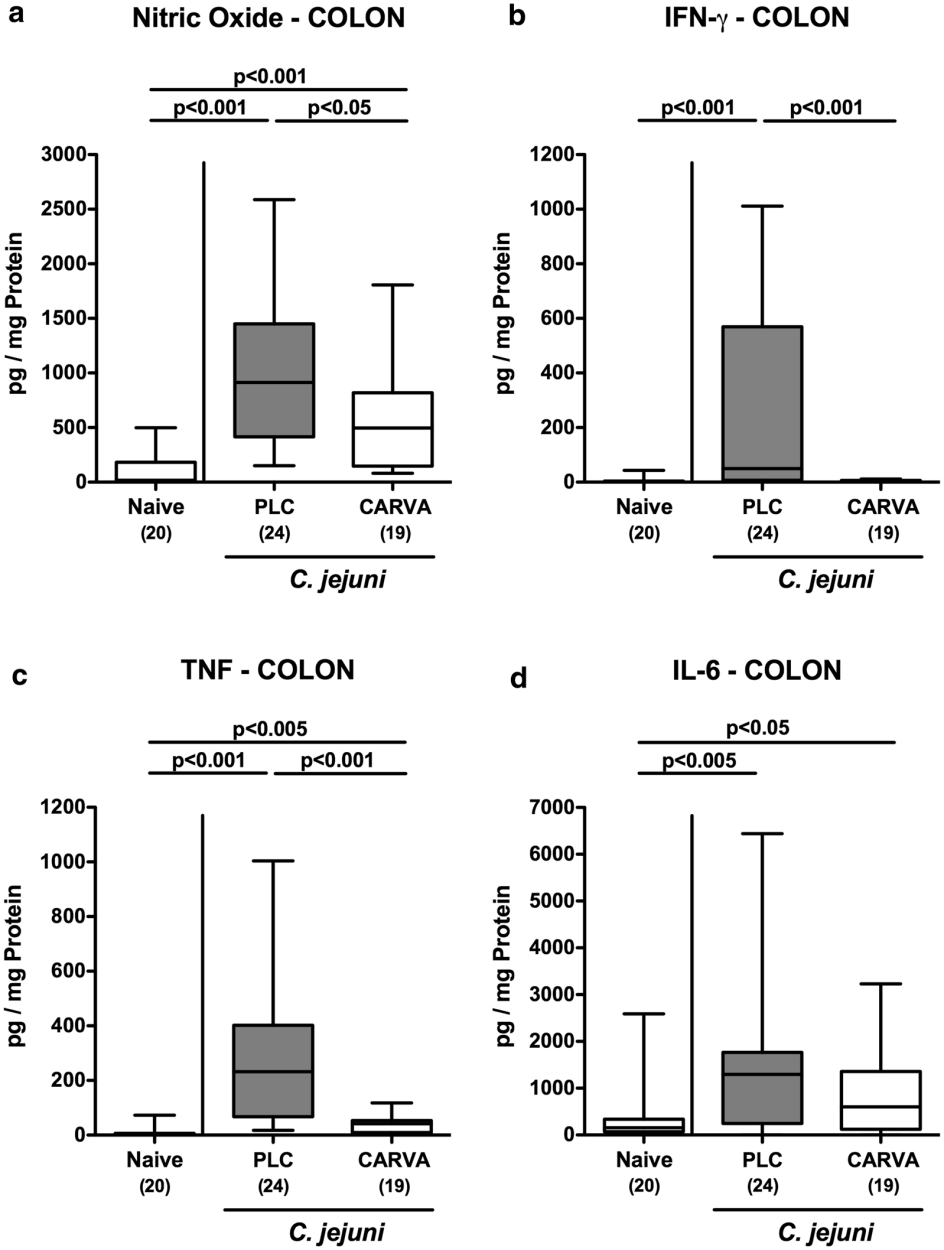
Colonic pro-inflammatory mediator secretion following carvacrol treatment of *C. jejuni* infected mice. Starting 4 days prior peroral *C. jejuni* infection on days 0 and 1, secondary abiotic IL-10^−/−^ mice were treated with synthetic carvacrol (CARVA; white boxes) or placebo (PLC; grey boxes) via the drinking water. **a** Nitric oxide, **b** IFN-γ, **c** TNF, and **d** IL-6 concentrations were measured in supernatants of colonic ex vivo biopsies derived at day 6 post-infection. Naive mice served as uninfected controls. The total range, significance levels (p-values) determined by the Mann–Whitney U test and numbers of analyzed animals (in parentheses) are indicated. Data were pooled from four independent experiments

We next quantitatively assessed large intestinal immune responses upon carvacrol treatment of *C. jejuni* infected mice. Within 6 days following *C. jejuni* infection, distinct immune cell populations such as CD3+ T lymphocytes cells and B220+  B lymphocytes had multifold increased in the colonic mucosa and lamina propria of mice from either cohort (p < 0.001; Fig. 3c, d; Additional file 2: Fig. S2C, D). Notably, colonic T lymphocytes, however, were lower following carvacrol as compared to PLC treatment at day 6 p.i. (p < 0.005, Fig. 3c; Additional file 2: Fig. S2C). The increased large intestinal abundances of immune cells upon *C. jejuni* infection were accompanied by enhanced secretion of pro-inflammatory mediators such as nitric oxide (NO), tumor necrosis factor (TNF) and interleukin (IL)-6 in colonic ex vivo biopsies obtained at day 6 p.i. (p < 0.05–0.001; Fig. 4a, c, d). Carvacrol treatment of *C. jejuni* infected mice, however, resulted in lower colonic NO, interferon (IFN)-γ and TNF concentrations as compared to PLC application (p < 0.05–0.001; Fig. 4a–c). Of note, IFN-γ levels measured in the large intestines of carvacrol treated mice at day 6 p.i. did not differ from those of naive control mice (n.s.; Fig. 4b). Hence, carvacrol dampened *C. jejuni* induced colonic inflammation.

### Ileal inflammatory immune responses upon carvacrol treatment of *C. jejuni* infected mice

In humans and murine infection models, *C. jejuni* induced intestinal inflammation is considered to primarily affect the large intestinal tract resulting in acute enterocolitis [9]. Nevertheless, we expanded our intestinal inflammatory survey of campylobacteriosis induced in secondary abiotic IL-10^−/−^ mice to the small intestines. At day 6 p.i., PLC, but not carvacrol treated mice displayed almost three time higher numbers of apoptotic ileal epithelial cells (p < 0.001 vs. naive; Additional file 3: Fig. S3A and Additional file 4: Fig. S4A), which was accompanied by increased B cell counts in the mucosa and lamina propria of *C. jejuni* infected mice from the PLC, but not carvacrol cohort (p < 0.001; Additional file 3: Fig. S3D and Additional file 4: Fig. S4D). In addition, irrespective of the treatment regimen, mice displayed increased numbers of proliferating ileal epithelial cells (p < 0.05–0.001; Additional file 3: Fig. S3B and Additional file 4: Fig. S4B) as well as of T lymphocytes (p < 0.001; Additional file 3: Fig. S3C and Additional file 4: Fig. S4C) in the small intestinal mucosa and lamina propria. The inflammation-ameliorating effects of carvacrol treatment also in the small intestinal tract of *C. jejuni* infected mice was further supported by increased secretion of pro-inflammatory cytokines such as TNF and IFN-γ in ileal ex vivo biopsies taken from PLC (p < 0.05–0.005 vs. naive), but not carvacrol treated mice at day 6 p.i. (p < 0.05 vs. PLC; Additional file 5: Fig. S5). Hence, the campylobacteriosis ameliorating properties of carvacrol were not restricted to the large intestinal tract, but also effective in the distal small intestines.

### Inflammatory immune responses in MLN upon carvacrol treatment of *C. jejuni* infected mice

**Fig. 5:**
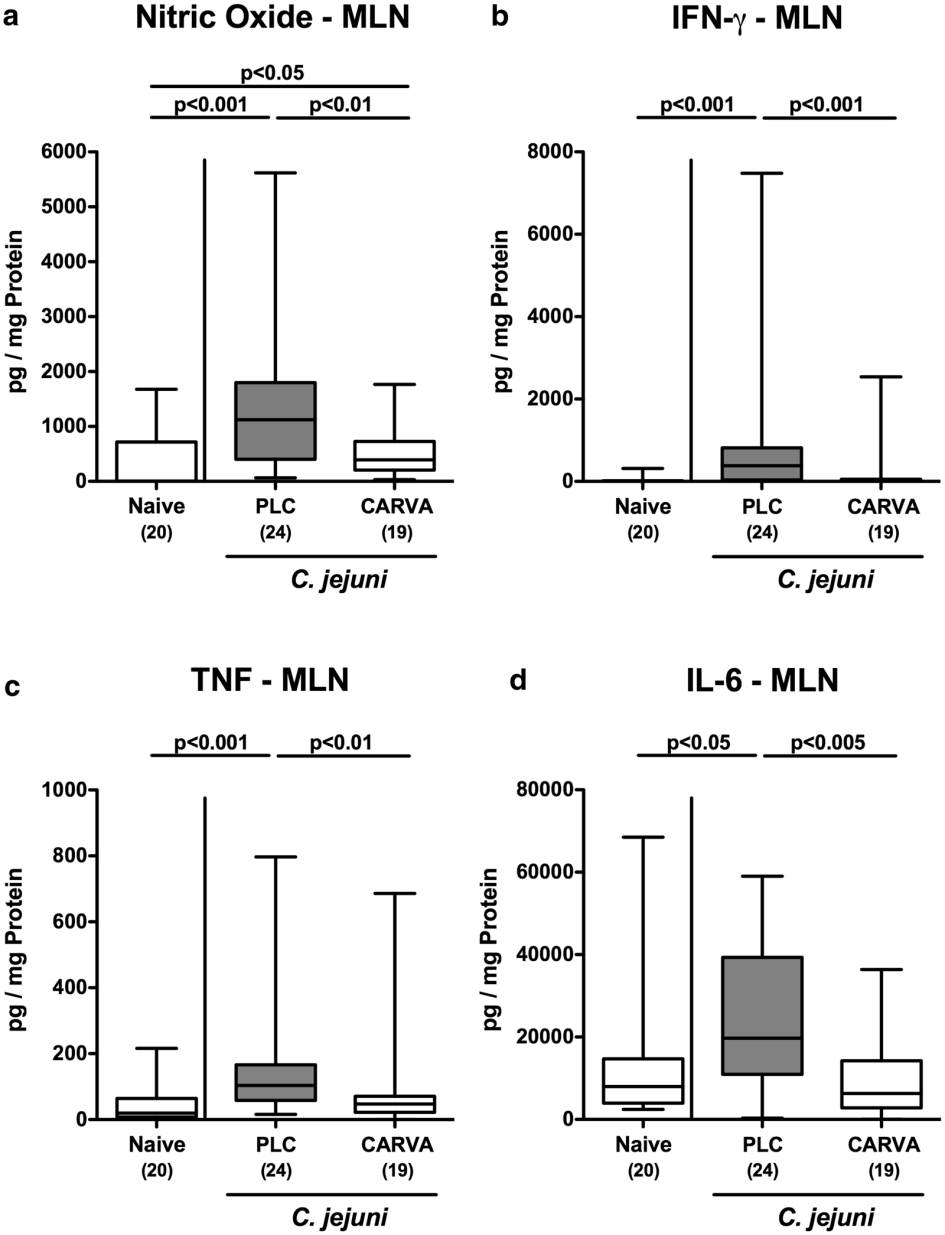
Pro-inflammatory mediator secretion in mesenteric lymph nodes following carvacrol treatment of *C. jejuni* infected mice. Starting 4 days prior peroral *C. jejuni* infection on days 0 and 1, secondary abiotic IL-10^−/−^ mice were treated with synthetic carvacrol (CARVA; white boxes) or placebo (PLC; grey boxes) via the drinking water. **a** Nitric oxide, **b** IFN-γ, **c** TNF, and **d** IL-6 concentrations were measured in supernatants of ex vivo biopsies derived from mesenteric lymph nodes (MLN) at day 6 post-infection. Naive mice served as uninfected controls. The total range, significance levels (p-values) determined by the Mann–Whitney U test and numbers of analyzed animals (in parentheses) are indicated. Data were pooled from four independent experiments

We next surveyed disease ameliorating effects of carvacrol treatment in the MLN of *C. jejuni* infected mice. In support of our results obtained from the colon and ileum, less distinct secretion of pro-inflammatory mediators such as NO, IFN-γ, TNF and IL-6 could be assessed in MLN of carvacrol as compared to PLC treated mice at day 6 p.i. (p < 0.01–0.001; Fig. 5). Of note, the concentrations of the three latter cytokines measured in carvacrol treated *C. jejuni* infected mice did not differ from those obtained from naive control animals (n.s.; Fig. 5b–d). Hence, the anti-inflammatory properties of carvacrol in *C. jejuni* infected mice were also effective in MLN draining the intestinal tract.

### Extra-intestinal including systemic inflammatory immune responses upon carvacrol treatment of *C. jejuni* infected mice

**Fig. 6:**
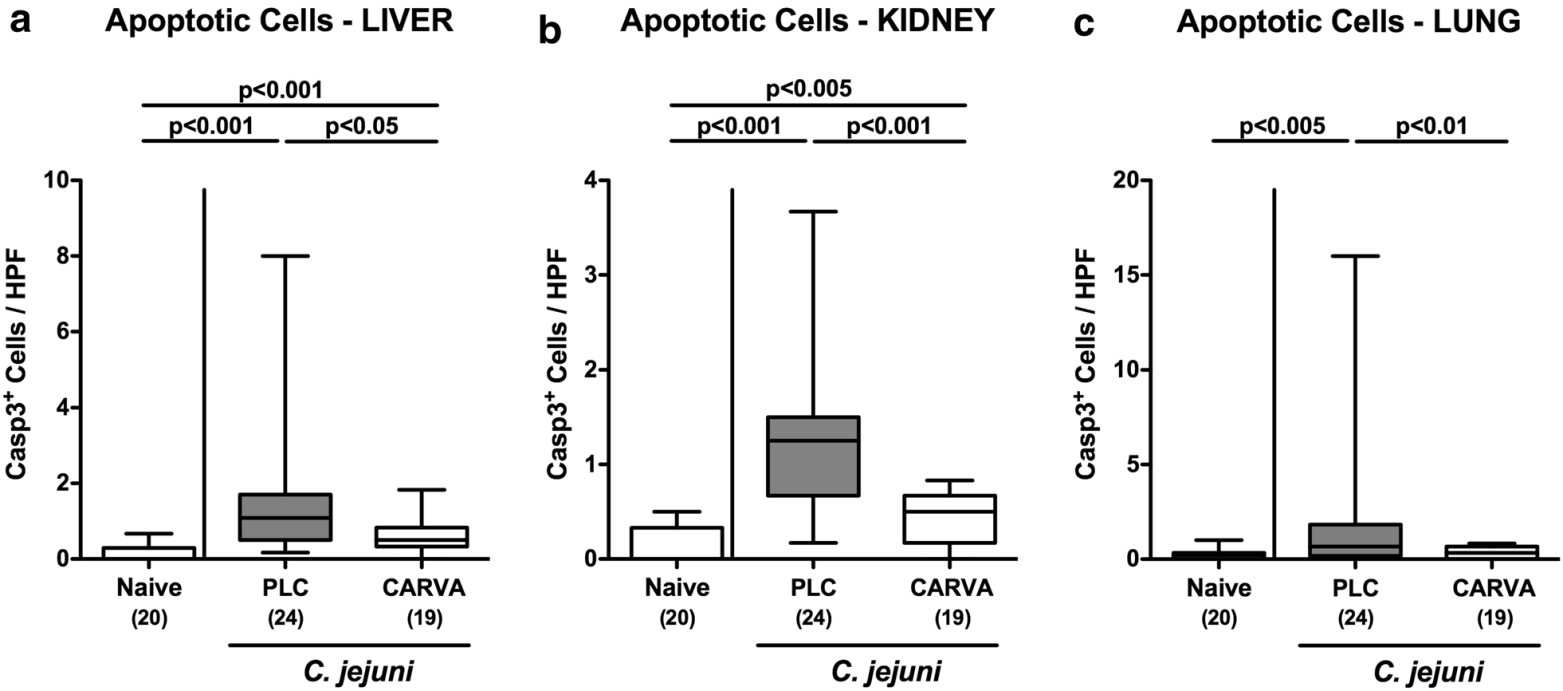
Extra-intestinal apoptosis following carvacrol treatment of *C. jejuni* infected mice. Starting 4 days prior peroral *C. jejuni* infection on days 0 and 1, secondary abiotic IL-10^−/−^ mice were treated with synthetic carvacrol (CARVA; white boxes) or placebo (PLC; grey boxes) via the drinking water. The average numbers of apoptotic cells (positive for caspase-3, Casp3) from six high power fields (HPF, ×400 magnification) per mouse were assessed microscopically in immunohistochemically stained paraffin sections derived from **a** liver, **b** kidneys and **c** lungs at day 6 post-infection. Naive mice served as uninfected controls. The total range, significance levels (p-values) determined by the Mann–Whitney U test and numbers of analyzed animals (in parentheses) are indicated. Data were pooled from four independent experiments

We further assessed disease-alleviating properties of carvacrol treatment in extra-intestinal including systemic compartments of *C. jejuni* infected mice. Increased numbers of caspase3 positive apoptotic cells could be observed in paraffin sections taken from liver, kidneys and lungs at day 6 p.i. (p < 0.005–0.001), whereas apoptotic cell counts in either organs were lower in carvacrol as compared to PLC treated mice (p < 0.05–0.001; Fig. 6; Additional file 6: Fig. S6). Of note, in lungs numbers of apoptotic cells did not differ in carvacrol treated *C. jejuni* infected and naive mice (n.s.; Fig. 6c, Additional file 6: Fig. S6C).

**Fig. 7:**
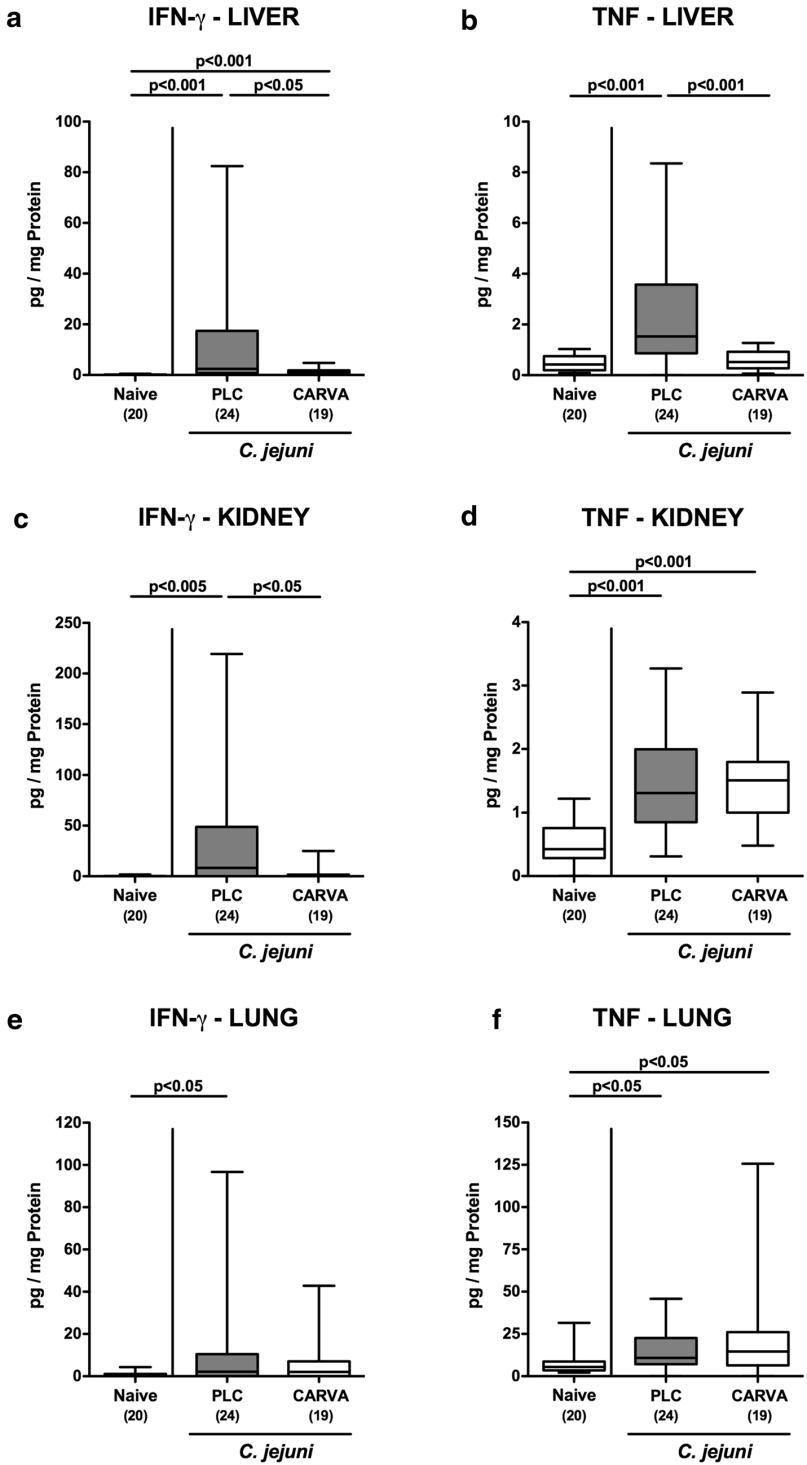
Extra-intestinal pro-inflammatory mediator secretion following carvacrol treatment of *C. jejuni* infected mice. Starting 4 days prior peroral *C. jejuni* infection on days 0 and 1, secondary abiotic IL-10^−/−^ mice were treated with synthetic carvacrol (CARVA; white boxes) or placebo (PLC; grey boxes) via the drinking water. **a**, **c**, **e** IFN-γ and **b**, **d**, **f** TNF concentrations were measured in supernatants of ex vivo biopsies derived from **a**, **b** liver, **c**, **d** kidneys and **e**, **f** lungs at day 6 post-infection. Naive mice served as uninfected controls. The total range, significance levels (p-values) determined by the Mann–Whitney U test and numbers of analyzed animals (in parentheses) are indicated. Data were pooled from four independent experiments

**Fig. 8:**
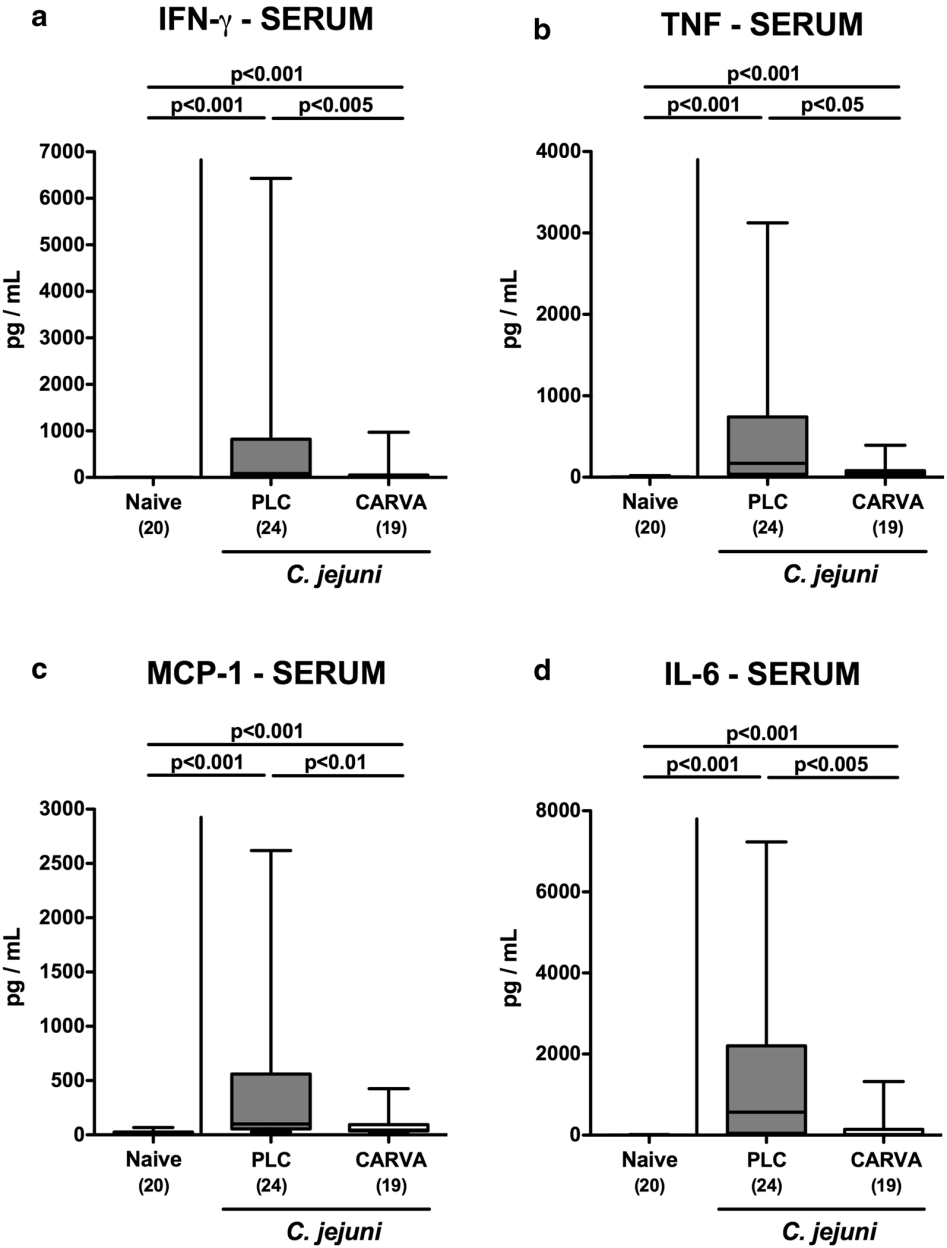
Systemic pro-inflammatory mediator secretion following carvacrol treatment of *C. jejuni* infected mice. Starting 4 days prior peroral *C. jejuni* infection on days 0 and 1, secondary abiotic IL-10^−/−^ mice were treated with synthetic carvacrol (CARVA; white boxes) or placebo (PLC; grey boxes) via the drinking water. **a** IFN-γ, **b** TNF, **c** MCP-1 and **d** IL-6 concentrations were measured serum samples taken at day 6 post-infection. Naive mice served as uninfected controls. The total range, significance levels (p-values) determined by the Mann–Whitney U test and numbers of analyzed animals (in parentheses) are indicated. Data were pooled from four independent experiments

We next measured pro-inflammatory cytokine secretion in respective ex vivo biopsies. At day 6 p.i., lower IFN-γ as well as TNF concentrations could be assessed in the liver of carvacrol as compared to PLC treated mice (p < 0.05 and p < 0.001, respectively; Fig. 7a, b), which also held true for renal IFN-γ protein levels (p < 0.05; Fig. 7c), but not TNF concentration (n.s.; Fig. 7d). Moreover, IFN-γ concentrations increased upon *C. jejuni* infection in the lungs of PLC (p < 0.05; Fig. 7e), but not carvacrol treated mice (n.s.; Fig. 7e), whereas elevated pulmonary TNF levels could be obtained at day 6 p.i., irrespective of the treatment regimen (p < 0.05; Fig. 7f). Remarkably, the inflammation-dampening effects of carvacrol in *C. jejuni* infected mice could also be observed systemically as indicated by lower increases in pro-inflammatory mediators such as IFN-γ, TNF, monocyte chemoattractant protein-1 (MCP-1) and IL-6 measured in serum samples taken from carvacrol as compared to PLC treated mice at day 6 p.i. (p < 0.05–0.005; Fig. 8). Hence, carvacrol treatment dampens *C. jejuni* induced pro-inflammatory responses also in extra-intestinal and even systemic compartments.

### Bacterial translocation into extra-intestinal including systemic compartments of carvacrol treated mice following *C. jejuni* infection

**Fig. 9:**
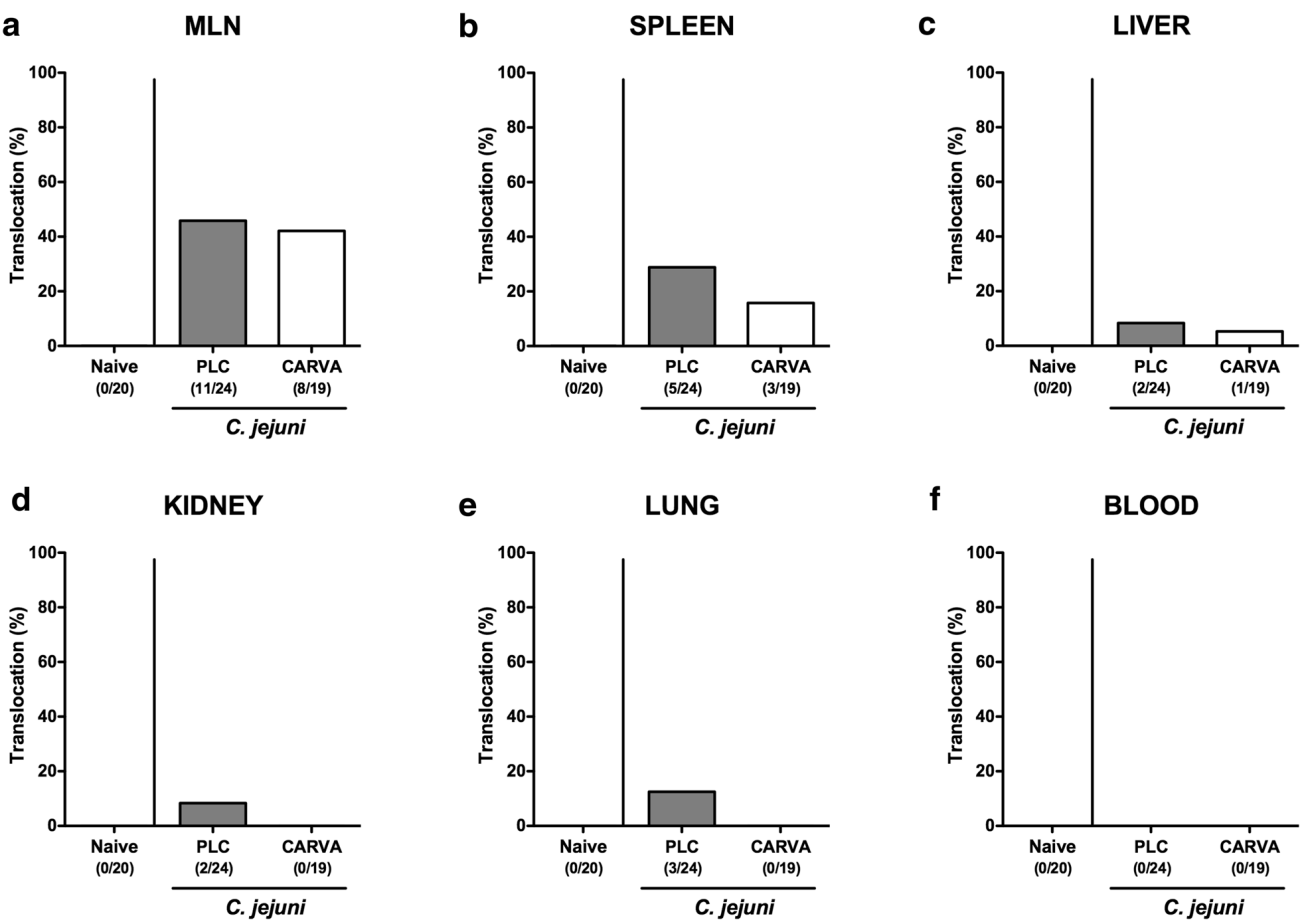
Bacterial translocation following carvacrol treatment of *C. jejuni* infected mice. Starting 4 days prior peroral *C. jejuni* infection on days 0 and 1, secondary abiotic IL-10^−/−^ mice were treated with synthetic carvacrol (CARVA; white boxes) or placebo (PLC; grey boxes) via the drinking water. At day 6 post-infection, the abundance of *C. jejuni* was assessed in ex vivo biopsies derived from **a** MLN, **b** spleen, **c** liver, **d** kidneys, **e** lungs and **f** cardiac blood by culture. The cumulative relative translocation rate of viable pathogens into the respective compartment out of four independent experiments is indicated (in %)

We finally addressed whether carvacrol treatment had an impact on the translocation of viable pathogens originating from the gastrointestinal tract to extra-intestinal including systemic tissue sites. Whereas *C. jejuni* could be cultured from MLN of PLC and carvacrol treated mice in 45.8 and 42.1% of cases at day 6 p.i., respectively (Fig. 9a), pathogenic translocation rates were lower in spleens (15.8% vs. 20.8%), livers (5.3% vs. 8.3%), kidneys (0% vs. 8.3%) and lungs (0% vs 12.5%) taken from mice of the carvacrol versus PLC cohorts (Fig. 9b–e). Of note, all blood cultures were *C. jejuni* negative (Fig. 9f). Hence, carvacrol treatment of *C. jejuni* infected mice is accompanied with less frequent translocation of viable pathogens from the intestinal tract to extra-intestinal compartments.

## Discussion

In the European Union the annual financial burden to the public health systems and to loss of individual health and productivity due to foodborne *C. jejuni* infections are estimated to account for 2.4 billion Euro [33]. Given the progressive emergence of human campylobacteriosis, identification of non-synthetic antibiotic molecules that exert both, potent anti-*Campylobacter* and anti-inflammatory effects in infected individuals is highly appreciable in order to combat pathogen-induced morbidities. The phenolic compound carvacrol has been shown to exert antimicrobial activities by increasing the membrane permeability of Gram-negative bacteria including *C. jejuni* [34] and thus, represents a promising option to replace conventionally used synthetic antibiotics for lowering the *C. jejuni* burden in livestock animals and avoiding the development of antibiotic resistant *C. jejuni* strains. In fact, the European Union has approved carvacrol as animal food supplement on the basis of results derived from carvacrol treatment studies in chicken farms in order to reduce the abundance of *Campylobacter* in poultry farming and meat production [35]. Notably, carvacrol constitutes a major component of oregano and thyme oils representing a “generally as safe recognized” phytoherbal compound [36–40] that has been pharmaceutically approved for the treatment of gastrointestinal morbidities in humans [41, 42]. However, scientifically validated data regarding carvacrol application against *C. jejuni* induced enterocolitis in humans are scarce.

In the present pre-clinical intervention study we therefore applied a well-established clinical *C. jejuni* infection model of acute campylobacteriosis by using secondary abiotic IL-10^−/−^ mice which display the clinical hallmarks of severe campylobacteriosis seen in human patients. In our study, we applied carvacrol via the drinking water in a concentration of 500 mg/l that was more than three times higher than the MIC_90_ value of 150 mg/l that had been determined in 20 *C. jejuni* insolates before. Carvacrol treatment starting 4 days prior murine infection could sufficiently lower intestinal *C. jejuni* burdens up to 2 orders of magnitude until day 6 p.i. The antimicrobial properties of carvacrol directed against food-borne pathogens such as *C. jejuni* [26, 29, 30, 37] *Salmonella* spp. [37, 43–45], *Escherichia coli* O157:H7 [46] and *Bacillus cereus* [47, 48] have been shown in vitro recently. Synthetic carvacrol was, however, more effective against Gram-negative bacteria than against Gram-positive species [49].

Remarkably, carvacrol treatment alleviated *C. jejuni* induced symptoms of campylobacteriosis including wasting and bloody diarrhea. Previous in vitro studies revealed that carvacrol reduced *C. jejuni* virulence by inhibition of motility, cell attachment, tissue invasion and toxin production and disrupted bacterial ATP production leading to bacterial cell death [22–27, 29, 30, 50]. In addition, carvacrol reduced *Campylobacter* colonization in chicken in vivo and inhibited biofilm formation [26]. Ameliorated murine campylobacteriosis was further characterized by less distinct *C. jejuni* induced apoptosis in both, colonic and ileal epithelia, whereas intestinal cell proliferative/regenerative properties counteracting pathogen-induced cell damage were enhanced upon carvacrol treatment. Furthermore, carvacrol application dampened intestinal pro-inflammatory immune responses upon *C. jejuni* infection as indicated by less abundance of T and B lymphocytes in the mucosa and lamina propria of both, the large and the small intestines and lower concentrations of pro-inflammatory mediators including TNF and IL-6 in the intestinal tract and further, in the MLN draining the infected intestines. These results are supported by recent in vitro studies showing that carvacrol treatment of both, stimulated dendritic cells and macrophages resulted in less distinct TNF and IFN-γ secretion [51], whereas carvacrol could down-regulate lipopolysaccharide (LPS) induced expression of pro-inflammatory cytokines such as TNF and IL-6 in broilers [52].

Notably, the inflammation-dampening properties of exogenous carvacrol was not restricted to the intestinal tract, but could also be observed in extra-intestinal organs such as liver, kidneys and lungs as indicated by less distinct apoptosis and secretion of the pro-inflammatory cytokines TNF and IFN-γ in respective organs. In support, carvacrol has been shown to improve survival during LPS-induced endotoxinemia and acute lung injury in mice and to result in less distinct secretion of pro-inflammatory cytokines including TNF and IL-6 [53].

Strikingly, the potent campylobacteriosis ameliorating effects upon carvacrol treatment could also be assessed systemically given that serum concentrations of TNF, IFN-γ, MCP-1 and IL-6 were lower in carvacrol as compared to PLC treated *C. jejuni* infected mice. Of note, translocation of viable pathogens from the intestinal tract to extra-intestinal organs occurred less frequently in the carvacrol versus PLC cohort. Even though all blood cultures were *C. jejuni* negative, one needs to take further into account that soluble bacterial molecules such as LOS and other cell wall constituents might have been transported via the circulation and been responsible for the deleterious outcome of severe campylobacteriosis in PLC control mice.

Overall, it is tempting to speculate that the observed disease-ameliorating properties of carvacrol are due to an orchestrated sum effect of distinct anti-inflammatory features of the compound: (i) lower intestinal pathogen loads and hence, (ii) less LOS exposure less distinctly inducing pro-inflammatory mediator secretion, (iii) hypothetical direct anti-LOS effect of carvacrol, (iv) interfering with distinct *C. jejuni* virulence factors by blocking motility, adhesion, invasion, LOS expression, leading to (v) less recruitment of immune cells resulting in (vi) less secretion of pro-inflammatory mediators, (vii) less cytotoxicity including apoptosis, (viii) more counter-regulatory cell proliferation/regeneration, (ix) less pathogenic translocation, and (x) less extra-intestinal including (xi) systemic immune responses.

Taken together these beneficial effects result in a significant amelioration of disease and better overall clinical outcome of mice in the clinical infection model for human campylobacteriosis.

## Conclusion

The lowered *C. jejuni* loads and alleviated symptoms observed in the here applied clinical murine model for human campylobacteriosis highlight the application of carvacrol as a promising option not only for the treatment of campylobacteriosis in humans and hence, for prevention of post-infectious sequelae, but also for the reduction of *C. jejuni* colonization in lifestock animals.

## Methods

### Determination of minimal inhibitory concentrations of carvacrol

For determination of minimal inhibitory concentration (MIC) values of carvacrol, 20 *C. jejuni* isolates including the reference strain 81–176 used for infection of mice (see below) were tested for their antimicrobial susceptibility applying the broth microdilution method. Procedures regarding inoculum density, growth medium, incubation time and conditions were performed in accordance with the recommendations given in the Clinical and Laboratory Standards Institute (CLSI) document VET01-Ed5. Twofold serial dilutions ranging from 0.008 to 8.0 mmol/l (1–1202 µg/ml) for carvacrol were tested. Stock solutions were prepared in Mueller–Hinton broth and adjusted to pH 7.3. The *C. jejuni* reference strain DSM 4688 was used for quality control purposes. The MIC value of the reference strain was tested in advance in three independent experiments using the broth microdilution method and the broth macrodilution method.

### Generation of secondary abiotic mice

IL-10^−/−^ mice (female and male, all in C57BL/6j background) were reared under specific pathogen free (SPF) conditions in the same unit of the Forschungseinrichtungen für Experimentelle Medizin (FEM, Charité—University Medicine Berlin). In order to counteract physiological colonization resistance and thus assure stable gastrointestinal *C. jejuni* colonization [13], secondary abiotic mice with a depleted gut microbiota were generated as described earlier [13, 54]. In brief, immediately post weaning 3-week-old mice were subjected to a 10-week course of broad-spectrum antibiotic treatment by adding ampicillin plus sulbactam (1 g/l; Ratiopharm, Germany), vancomycin (500 mg/l; Cell Pharm, Germany), ciprofloxacin (200 mg/l; Bayer Vital, Germany), imipenem (250 mg/l; MSD, Germany) and metronidazole (1 g/l; Fresenius, Germany) to the autoclaved drinking water (ad libitum). Two days before pathogenic challenge the antibiotic cocktail was replaced by autoclaved tap water to assure antibiotic washout.

### Carvacrol treatment

Four days prior *C. jejuni* infection treatment with carvacrol (Sigma-Aldrich, Munich, Germany; daily dose of 100 mg carvacrol per kg body weight) was initiated by dissolving the compound in Tween 80 (0.2% v/v) to a final concentration of 500 mg/l autoclaved tap water (ad libitum). Placebo control mice received Tween 80 only.

### *Campylobacter jejuni* infection

Twelve-week old mice were perorally challenged with 10^9^ colony forming units (CFU) of the highly pathogenic *C. jejuni* reference strain 81–176 by gavage (in a total volume of 0.3 ml phosphate buffered saline (PBS), Gibco, Life Technologies, UK). Animals were continuously maintained in a sterile environment (autoclaved food and drinking water or sterile antibiotic cocktail) and handled under strict aseptic conditions in order to avoid contaminations.

### Clinical conditions

Before and after *Campylobacter jejuni* infection clinical conditions of mice were assessed on a daily basis applying a standardized cumulative clinical score (maximum 12 points) addressing the abundance of blood in feces (0: no blood; 2: microscopic detection of blood by the Guajac method using Haemoccult, Beckman Coulter/PCD, Germany; 4: macroscopic blood visible), diarrhea (0: formed feces; 2: pasty feces; 4: liquid feces), and the clinical aspect (0: normal; 2: ruffled fur, less locomotion; 4: isolation, severely compromised locomotion, pre-final aspect) as described earlier [55].

### Sampling procedures

At day 6 p.i., mice were sacrificed by isofluran inhalation (Abbott, Germany). Luminal gastrointestinal samples (i.e., from stomach, duodenum, ileum and colon) and ex vivo biopsies were taken from colon, mesenteric lymph nodes (MLN), liver, kidneys, lungs, and spleen under sterile conditions. Intestinal samples were collected from each mouse in parallel for microbiological, immunohistopathological and immunological analyses. The absolute colonic lengths were measured with a ruler (in cm).

### Pathogenic colonization and translocation

*Campylobacter jejuni* loads were surveyed in fecal samples over time p.i., and upon necropsy in luminal samples taken from the stomach, duodenum, ileum and colon as well as in homogenates of ex vivo biopsies derived from MLN, spleen, liver, kidney and lung as well as in cardiac blood samples by culture as described previously [13, 56]. In brief, intraluminal gastrointestinal samples and respective ex vivo biopsies were homogenized in sterile PBS with a pistil and serial dilutions plated onto karmali agar (Oxoid, Wesel, Germany) and incubated in a microaerophilic atmosphere for at least 48 h. Cardiac blood (0.2 ml) was immediately streaked onto karmali agar plates. The detection limit of viable pathogens was approximately 100 CFU/g.

### Immunohistochemistry

In situ immunohistochemical analyses were performed in ex vivo biopsies derived from colon, ileum, liver, kidneys, and lungs that had been immediately fixed in 5% formalin and embedded in paraffin as stated elsewhere [32, 57–59]. In brief, in order to detect apoptotic epithelial cells, proliferation epithelial cells, T lymphocytes, and B lymphocytes, paraffin sections (5 μm) were stained with primary antibodies directed against cleaved caspase 3 (Asp175, Cell Signaling, Beverly, MA, USA, 1:200), Ki67 (TEC3, Dako, Denmark, 1:100), CD3 (#N1580, Dako, 1:10), and B220 (No. 14–0452-81, eBioscience; 1:200), respectively. After incubation with the primary antibody (30 min), sections were incubated for another 30 min with the respective secondary antibody (for anti-cleaved caspase 3 and anti-CD3 staining: biotinylated donkey anti-rabbit antibody; for anti-Ki67 and anti-B220 staining: biotinylated rabbit anti-rat antibody; all purchased from Dianova, Hamburg, Germany). The Streptavidin–Alkaline Phosphatase Kit (Dako) using Fast Red as chromogen was applied as detection system. Negative controls were generated in samples in which the respective primary antibody had been omitted. Positively stained cells were then examined by light microscopy (magnification 100× and 400×), and for each mouse the average number of respective positively stained cells was determined within at least six high power fields (HPF, 0.287 mm^2^, 400× magnification) by a blinded independent investigator.

### Pro-inflammatory mediators

Colonic and ileal ex vivo biopsies were cut longitudinally and washed in PBS. Ex vivo biopsies derived from liver (approximately 1 cm^3^), kidney (one half after longitudinal cut), lung, MLN (3–4 lymph nodes) or strips of approximately 1 cm^2^ colonic or ileal tissues were placed in 24-flat-bottom well culture plates (Nunc, Germany) containing 500 μl serum-free RPMI 1640 medium (Gibco, life technologies, UK) supplemented with penicillin (100 U/ml) and streptomycin (100 µg/ml; PAA Laboratories, Germany). After 18 h at 37 °C, culture supernatants and serum samples were tested for IFN-γ, TNF, IL-6, and MCP-1 by the Mouse Inflammation Cytometric Bead Assay (CBA; BD Biosciences, Germany) on a BD FACSCanto II flow cytometer (BD Biosciences). Systemic pro-inflammatory cytokine concentrations were measured in serum samples. NO concentrations were assessed by the Griess reaction [54, 60].

### Statistical analysis

Medians and levels of significance were determined using Mann–Whitney test (GraphPad Prism v7, USA) as indicated. Two-sided probability (p) values ≤ 0.05 were considered significant. Experiments were reproduced three times.

## Additional files

**Additional file 1: Figure S1.** Kinetic survey of clinical conditions following carvacrol treatment of *C. jejuni* infected mice. Starting 4 days prior peroral *C. jejuni* infection on day (d) 0 and d1, secondary abiotic IL-10^−/−^ mice were treated with synthetic carvacrol (CARVA; white boxes) or placebo (PLC; grey boxes) via the drinking water. Severities of clinical symptoms were surveyed daily from d0 until d6 applying a standardized clinical scoring system postinfection (see “Methods”). Box plots represent the 75th and 25th percentiles of medians (black bar inside the boxes). Total range, significance levels (p-values; determined between groups at respective time points; ***p < 0.001) by the Mann–Whitney U test and numbers of analyzed animals (in parentheses) are indicated. Data were pooled from four independent experiments.

**Additional file 2: Figure S2.** Representative photomicrographs illustrating apoptotic and proliferating epithelial as well as immune cells responses in large intestines upon carvacrol treatment of *C. jejuni* infected mice. Starting 4 days prior peroral *C. jejuni* infection on days 0 and 1, secondary abiotic IL-10^−/−^ mice were treated with synthetic carvacrol (CARVA) or placebo (PLC) via the drinking water. Naive mice served as uninfected controls. Photomicrographs reepresentative for four independent experiments illustrate the average numbers of (**A**) apoptotic epithelial cells (Casp3+), (**B**) proliferating epithelial cells (Ki67+), (**C**) T lymphocytes (CD3+), and (**D**) B lymphocytes (B220+) in at least six high power fields (HPF) as quantitatively assessed in colonic paraffin sections applying in situ immunohistochemistry at day 6 post-infection (**A**: 400× magnification, scale bar 20 μm; **B**–**D**: 100× magnification, scale bar 100 μm).

**Additional file 3: Figure S3.** Ileal apoptotic, proliferative and immune cell responses upon carvacrol treatment of *C. jejuni* infected mice. Starting 4 days prior peroral *C. jejuni* infection on days 0 and 1, secondary abiotic IL-10^−/−^ mice were treated with synthetic carvacrol (CARVA; white boxes) or placebo (PLC; grey boxes) via the drinking water. The average numbers of (**A**) apoptotic (positive for caspase3, Casp3) and (**B**) proliferative/regenerative (positive for Ki67) ileal epithelial cells as well as of (**C**) T lymphocytes (positive for CD3) and (**D**) B lymphocytes (positive for B220) in the ileal mucosa and lamina propria from six high power fields (HPF, 400× magnification) per mouse were assessed microscopically in immunohistochemically stained small intestinal paraffin sections at day 6 post-infection. Naive mice served as uninfected controls. The total range, significance levels (p-values) determined by the Mann–Whitney U test and numbers of analyzed animals (in parentheses) are indicated. Data were pooled from four independent experiments.

**Additional file 4: Figure S4.** Representative photomicrographs illustrating apoptotic and proliferating epithelial as well as immune cells responses in large intestines upon carvacrol treatment of *C. jejuni* infected mice. Starting 4 days prior peroral *C. jejuni* infection on days 0 and 1, secondary abiotic IL-10^−/−^ mice were treated with synthetic carvacrol (CARVA) or placebo (PLC) via the drinking water. Naive mice served as uninfected controls. Photomicrographs reepresentative for four independent experiments illustrate the average numbers of (**A**) apoptotic epithelial cells (Casp3+), (**B**) proliferating epithelial cells (Ki67+), (**C**) T lymphocytes (CD3+), and (**D**) B lymphocytes (B220+) in at least six high power fields (HPF) as quantitatively assessed in ileal paraffin sections applying in situ immunohistochemistry at day 6 post-infection (**A**: 400× magnification, scale bar 20 μm; **B**–**D**: 100× magnification, scale bar 100 μm).

**Additional file 5: Figure S5.** Ileal pro-inflammatory mediator secretion in carvacrol treated mice following *C. jejuni* infection. Starting 4 days prior peroral *C. jejuni* infection on days 0 and 1, secondary abiotic IL-10^−/−^ mice were treated with synthetic carvacrol (CARVA; white boxes) or placebo (PLC; grey boxes) via the drinking water. (**A**) IFN-γ and (**B**) TNF concentrations were measured in supernatants of ileal ex vivo biopsies derived at day 6 post-infection. Naive mice served as uninfected controls. The total range, significance levels (p-values) determined by the Mann–Whitney U test and numbers of analyzed animals (in parentheses) are indicated. Data were pooled from four independent experiments.

**Additional file 6: Figure S6.** Representative photomicrographs illustrating apoptotic cells responses in extra-intestinal compartments upon carvacrol treatment of *C. jejuni* infected mice. Starting 4 days prior peroral *C. jejuni* infection on days 0 and 1, secondary abiotic IL-10^−/−^ mice were treated with synthetic carvacrol (CARVA) or placebo (PLC) via the drinking water. Naive mice served as uninfected controls. Photomicrographs reepresentative for four independent experiments illustrate the average numbers of apoptotic cells (Casp3+) in (**A**) liver, (**B**) kidney and (**C**) lung in at least six high power fields (HPF) as quantitatively assessed in paraffin sections of respective ex vivo biopsies applying in situ immunohistochemistry at day 6 post-infection (100× magnification, scale bar 100 μm).

## Data Availability

Not applicable.

## Abbreviations

ATP: adenosine triphosphate
CARVA: carvacrol
CBA: Cytometric Bead Array
CFU: colony forming units
HPF: high power field
IFN: interferon
IL: interleukin
LOS: lipooligosaccharide
LPS: lipopolysaccharide
MCP-1: monocyte chemoattractant protein-1
MIC: minimal inhibitory concentration
MLN: mesenteric lymph nodes
NO: nitric oxide
PBS: phosphate-buffered saline
PLC: placebo
p.i.: post-infection
SPF: specific pathogen free
TLR: Toll-like Receptor
TNF: tumor necrosis factor
