# Supplementary figures and images for: Carvacrol ameliorates acute campylobacteriosis in a clinical murine infection model
Source: Gut Pathog. 2020 Jan 8;12:2. doi: 10.1186/s13099-019-0343-4 (PMC6947993; doi:10.1186/s13099-019-0343-4)

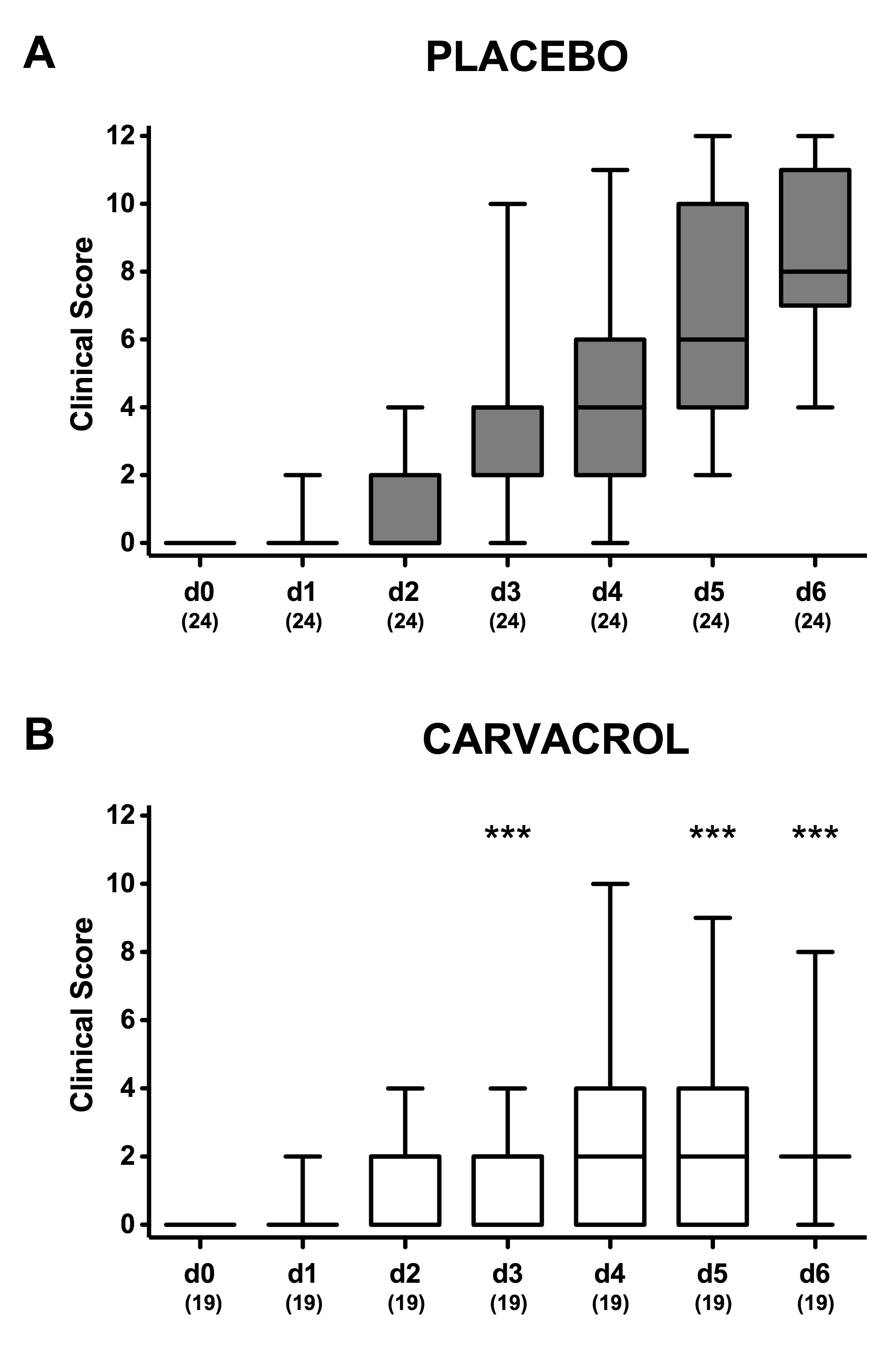

Supplement: Supplementary file 1 — Additional file 1: Figure S1. Kinetic survey of clinical conditions following carvacrol treatment of C. jejuni infected mice. Starting 4 days prior peroral C. jejuni infection on day (d) 0 and d1, secondary abiotic IL-10−/− mice were treated with synthetic carvacrol (CARVA; white boxes) or placebo (PLC; grey boxes) via the drinking water. Severities of clinical symptoms were surveyed daily from d0 until d6 applying a standardized clinical scoring system postinfection (see “Methods”). Box plots represent the 75th and 25th percentiles of medians (black bar inside the boxes). Total range, significance levels (p-values; determined between groups at respective time points; ***p < 0.001) by the Mann–Whitney U test and numbers of analyzed animals (in parentheses) are indicated. Data were pooled from four independent experiments. [file 13099_2019_343_MOESM1_ESM.tiff]

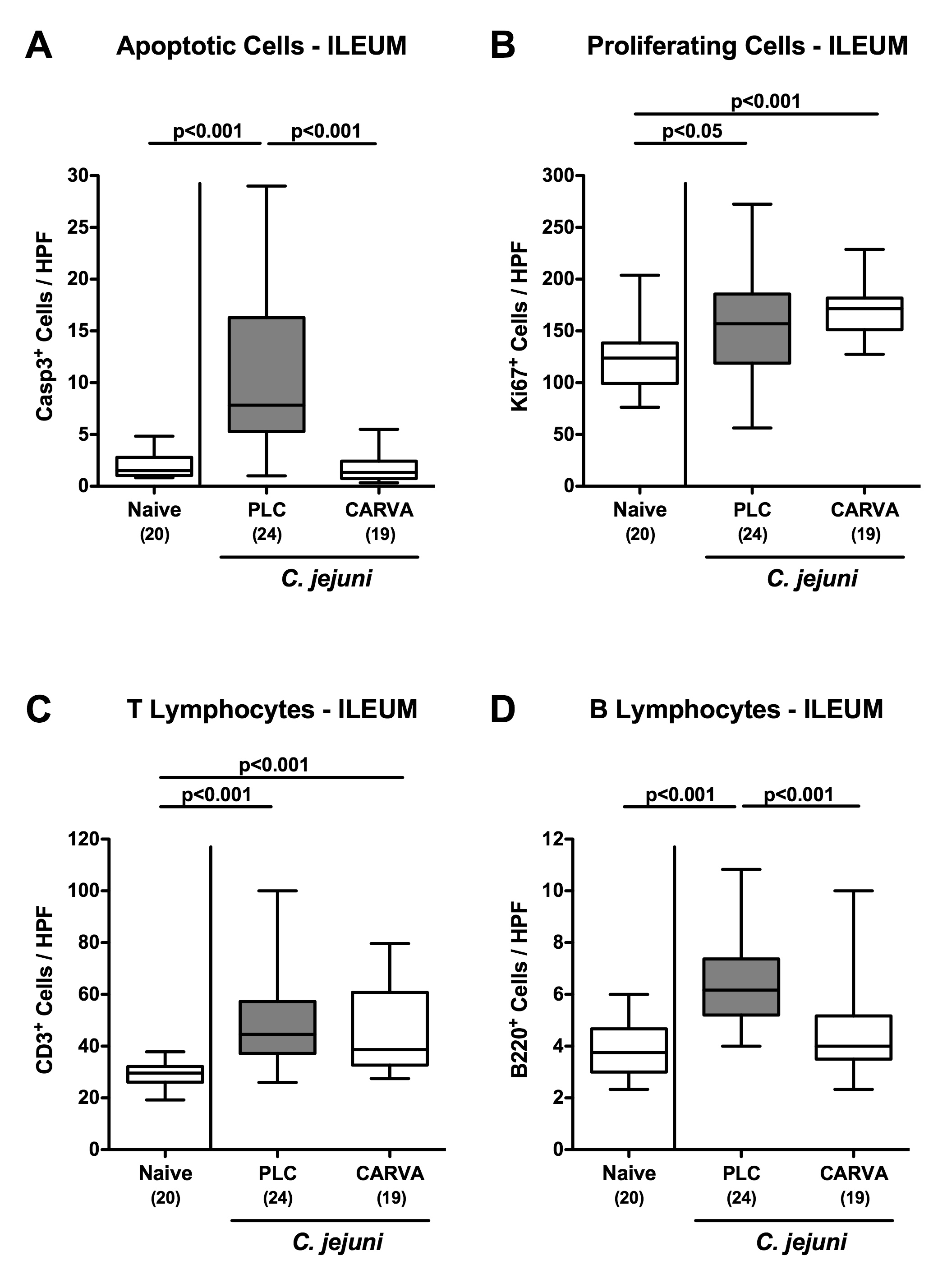

Supplement: Supplementary file 3 — Additional file 3: Figure S3. Ileal apoptotic, proliferative and immune cell responses upon carvacrol treatment of C. jejuni infected mice. Starting 4 days prior peroral C. jejuni infection on days 0 and 1, secondary abiotic IL-10−/− mice were treated with synthetic carvacrol (CARVA; white boxes) or placebo (PLC; grey boxes) via the drinking water. The average numbers of (A) apoptotic (positive for caspase3, Casp3) and (B) proliferative/regenerative (positive for Ki67) ileal epithelial cells as well as of (C) T lymphocytes (positive for CD3) and (D) B lymphocytes (positive for B220) in the ileal mucosa and lamina propria from six high power fields (HPF, 400× magnification) per mouse were assessed microscopically in immunohistochemically stained small intestinal paraffin sections at day 6 post-infection. Naive mice served as uninfected controls. The total range, significance levels (p-values) determined by the Mann–Whitney U test and numbers of analyzed animals (in parentheses) are indicated. Data were pooled from four independent experiments. [file 13099_2019_343_MOESM3_ESM.tiff]

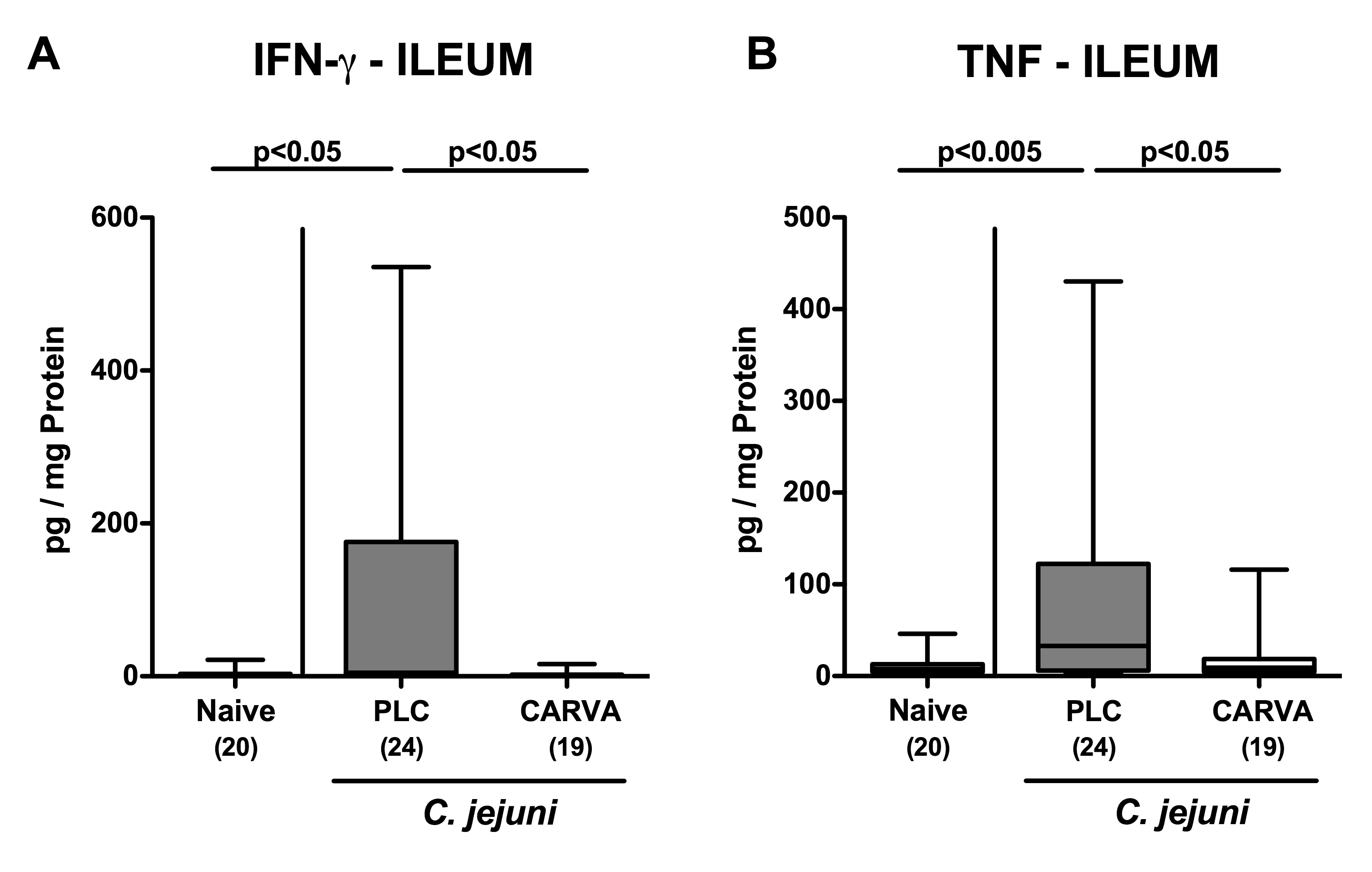

Supplement: Supplementary file 5 — Additional file 5: Figure S5. Ileal pro-inflammatory mediator secretion in carvacrol treated mice following C. jejuni infection. Starting 4 days prior peroral C. jejuni infection on days 0 and 1, secondary abiotic IL-10−/− mice were treated with synthetic carvacrol (CARVA; white boxes) or placebo (PLC; grey boxes) via the drinking water. (A) IFN-γ and (B) TNF concentrations were measured in supernatants of ileal ex vivo biopsies derived at day 6 post-infection. Naive mice served as uninfected controls. The total range, significance levels (p-values) determined by the Mann–Whitney U test and numbers of analyzed animals (in parentheses) are indicated. Data were pooled from four independent experiments. [file 13099_2019_343_MOESM5_ESM.tiff]
