# Supplementary material for: Carvacrol ameliorates acute campylobacteriosis in a clinical murine infection model
Source: Gut Pathog. 2020 Jan 8;12:2. doi: 10.1186/s13099-019-0343-4 (PMC6947993; doi:10.1186/s13099-019-0343-4)
Supplement: Supplementary file 2 — Additional file 2: Figure S2. Representative photomicrographs illustrating apoptotic and proliferating epithelial as well as immune cells responses in large intestines upon carvacrol treatment of C. jejuni infected mice. Starting 4 days prior peroral C. jejuni infection on days 0 and 1, secondary abiotic IL-10−/− mice were treated with synthetic carvacrol (CARVA) or placebo (PLC) via the drinking water. Naive mice served as uninfected controls. Photomicrographs reepresentative for four independent experiments illustrate the average numbers of (A) apoptotic epithelial cells (Casp3+), (B) proliferating epithelial cells (Ki67+), (C) T lymphocytes (CD3+), and (D) B lymphocytes (B220+) in at least six high power fields (HPF) as quantitatively assessed in colonic paraffin sections applying in situ immunohistochemistry at day 6 post-infection (A: 400× magnification, scale bar 20 μm; B–D: 100× magnification, scale bar 100 μm). [file 13099_2019_343_MOESM2_ESM.pptx]

## Slide 1
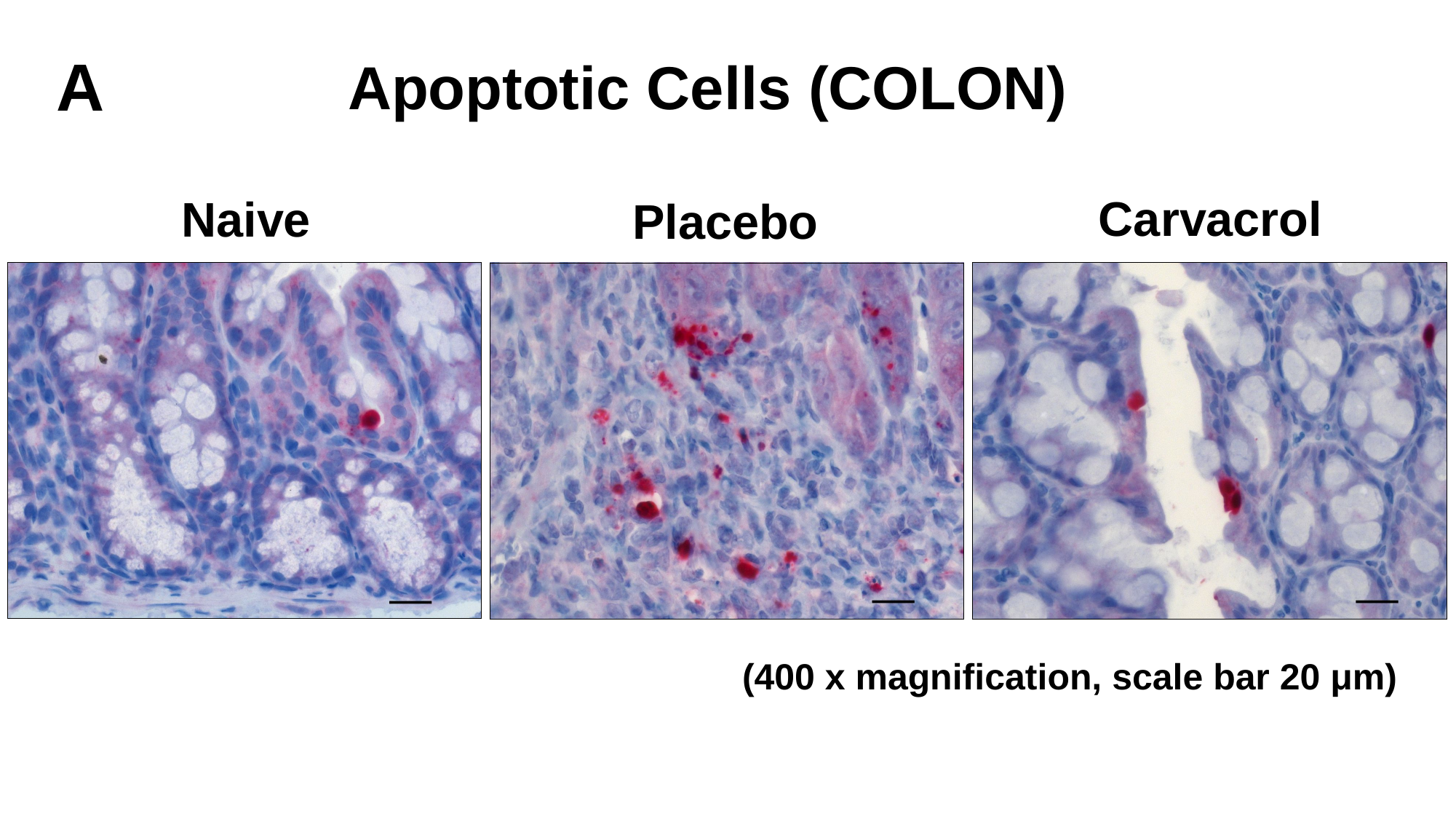

A
Apoptotic Cells (COLON)
Carvacrol
Naive
Placebo
(400 x magnification, scale bar 20 μm)

## Slide 2
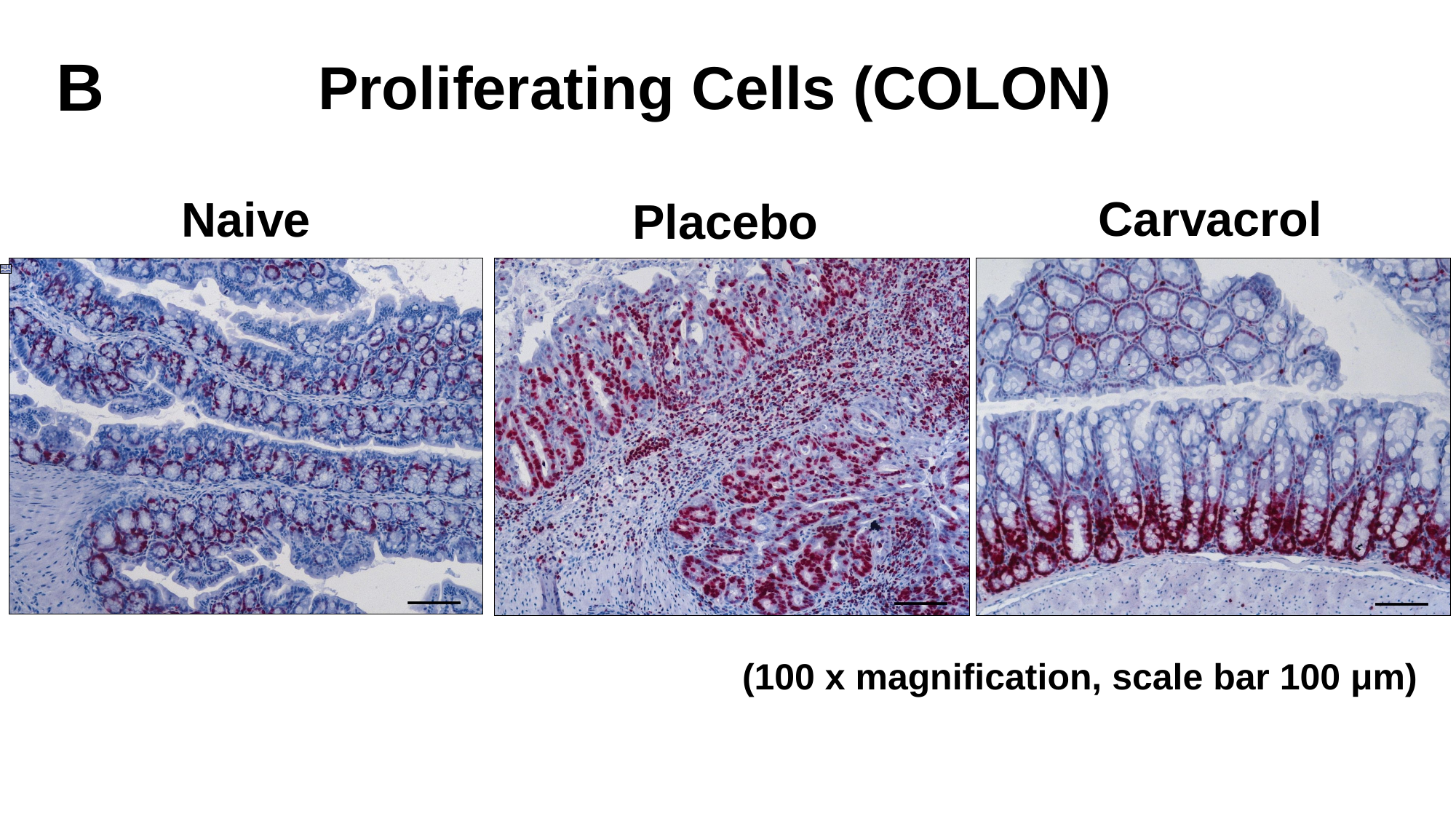

B
Proliferating Cells (COLON)
Carvacrol
Naive
Placebo
(100 x magnification, scale bar 100 μm)

## Slide 3
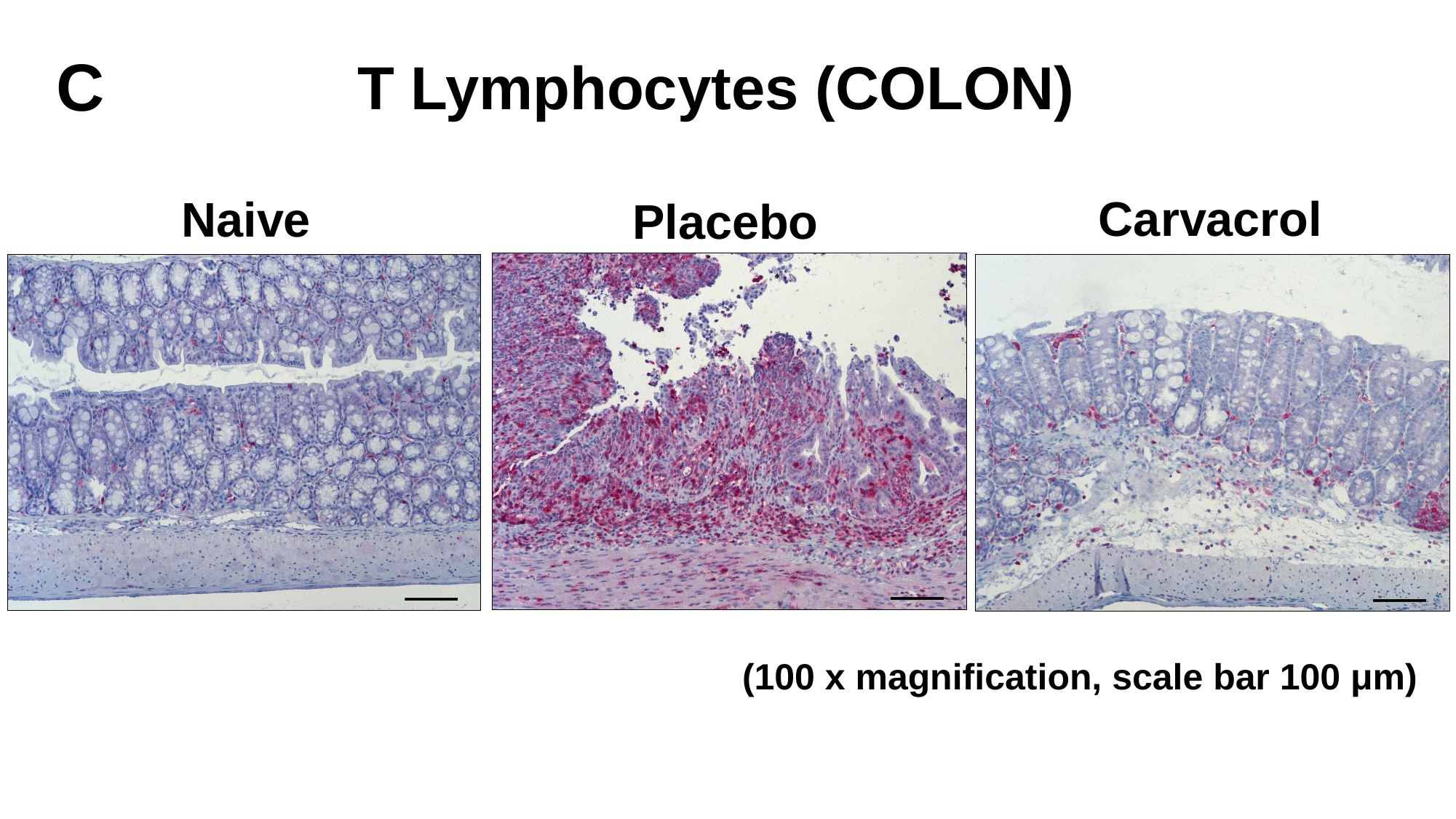

C
T Lymphocytes (COLON)
Carvacrol
Naive
Placebo
(100 x magnification, scale bar 100 μm)

## Slide 4
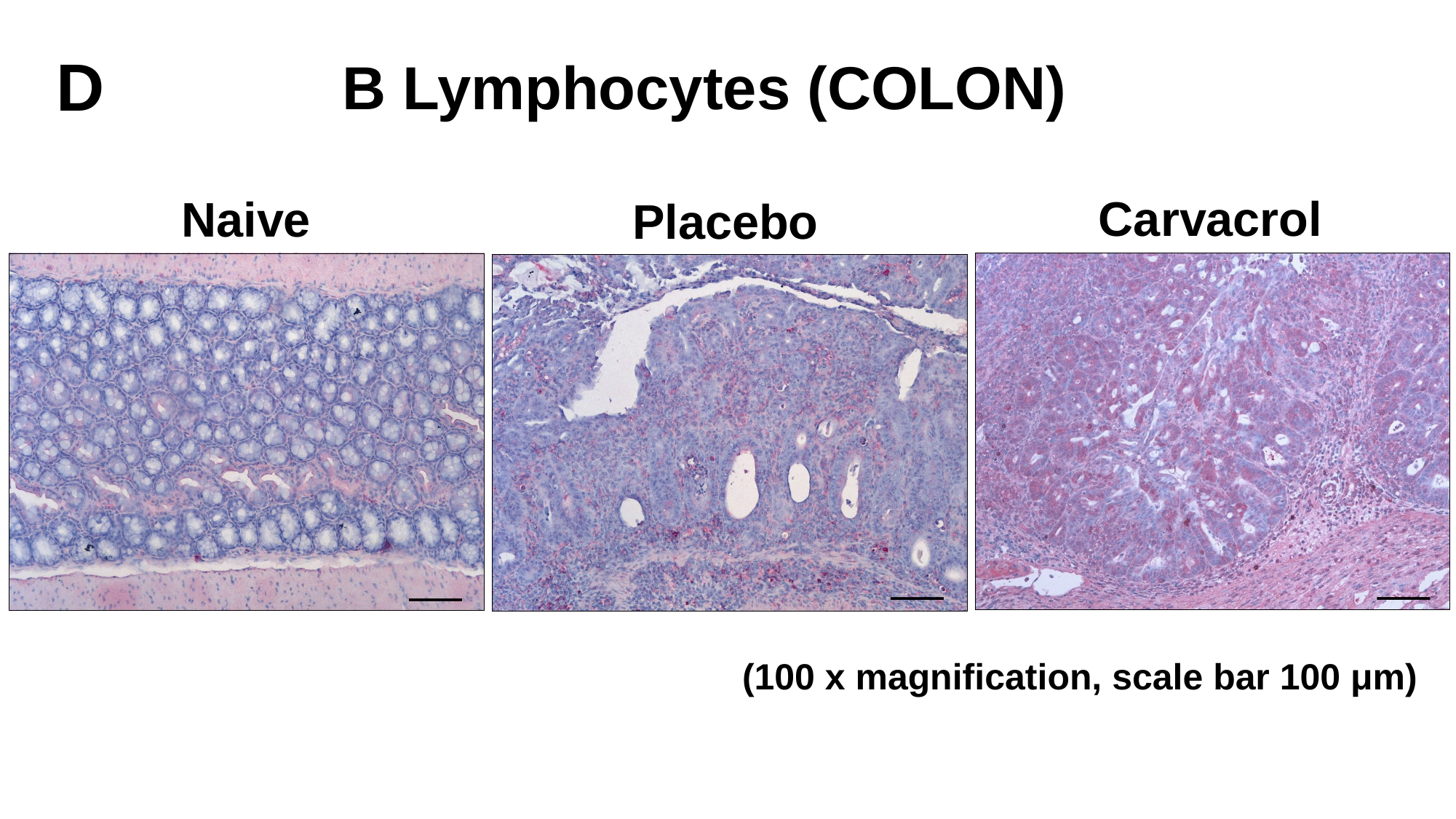

D
B Lymphocytes (COLON)
Carvacrol
Naive
Placebo
(100 x magnification, scale bar 100 μm)
