# Supplementary material for: Carvacrol ameliorates acute campylobacteriosis in a clinical murine infection model
Source: Gut Pathog. 2020 Jan 8;12:2. doi: 10.1186/s13099-019-0343-4 (PMC6947993; doi:10.1186/s13099-019-0343-4)
Supplement: Supplementary file 6 — Additional file 6: Figure S6. Representative photomicrographs illustrating apoptotic cells responses in extra-intestinal compartments upon carvacrol treatment of C. jejuni infected mice. Starting 4 days prior peroral C. jejuni infection on days 0 and 1, secondary abiotic IL-10−/− mice were treated with synthetic carvacrol (CARVA) or placebo (PLC) via the drinking water. Naive mice served as uninfected controls. Photomicrographs reepresentative for four independent experiments illustrate the average numbers of apoptotic cells (Casp3+) in (A) liver, (B) kidney and (C) lung in at least six high power fields (HPF) as quantitatively assessed in paraffin sections of respective ex vivo biopsies applying in situ immunohistochemistry at day 6 post-infection (100× magnification, scale bar 100 μm). [file 13099_2019_343_MOESM6_ESM.pptx]

## Slide 1
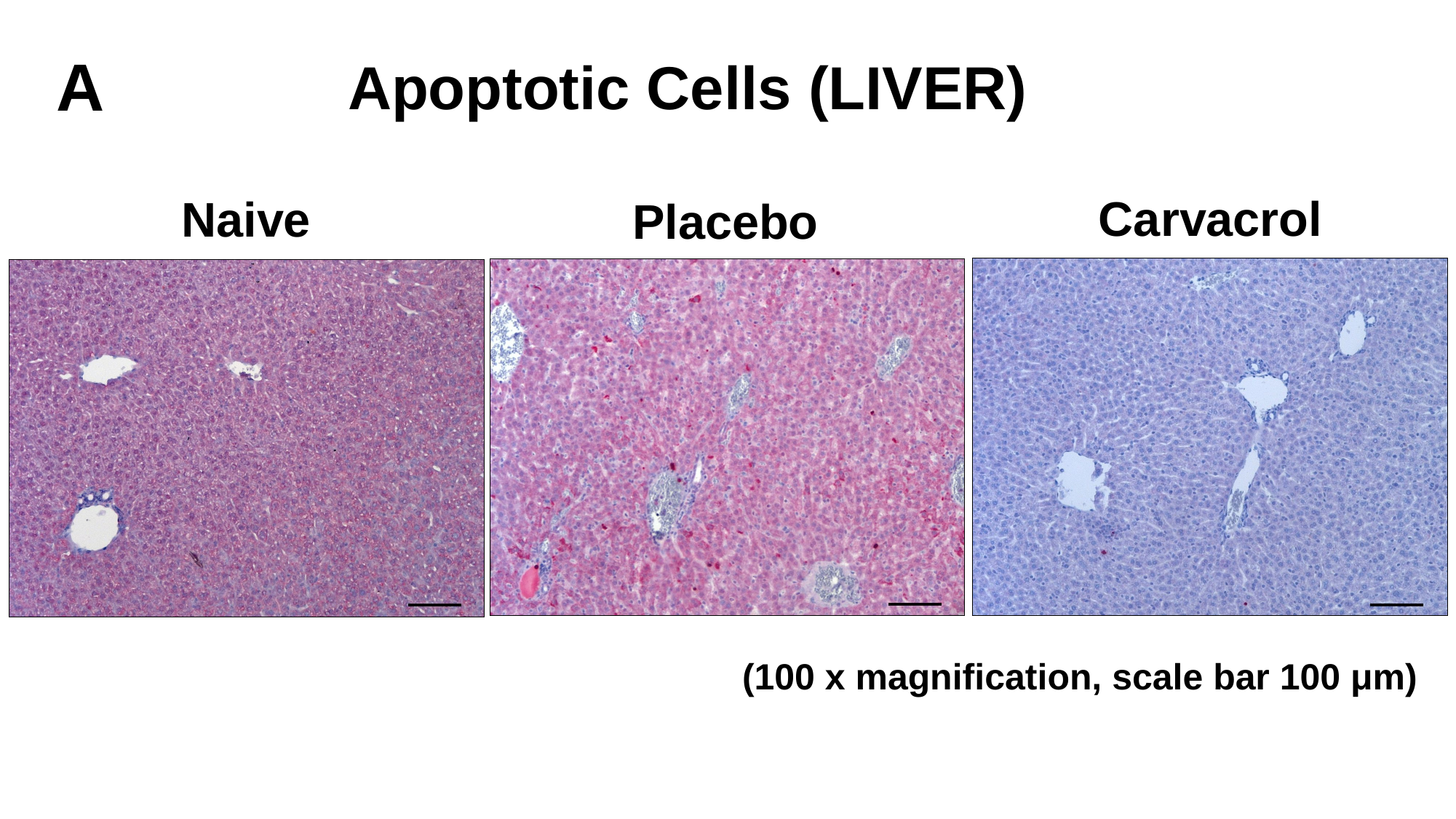

A
Apoptotic Cells (LIVER)
Carvacrol
Naive
Placebo
(100 x magnification, scale bar 100 μm)

## Slide 2
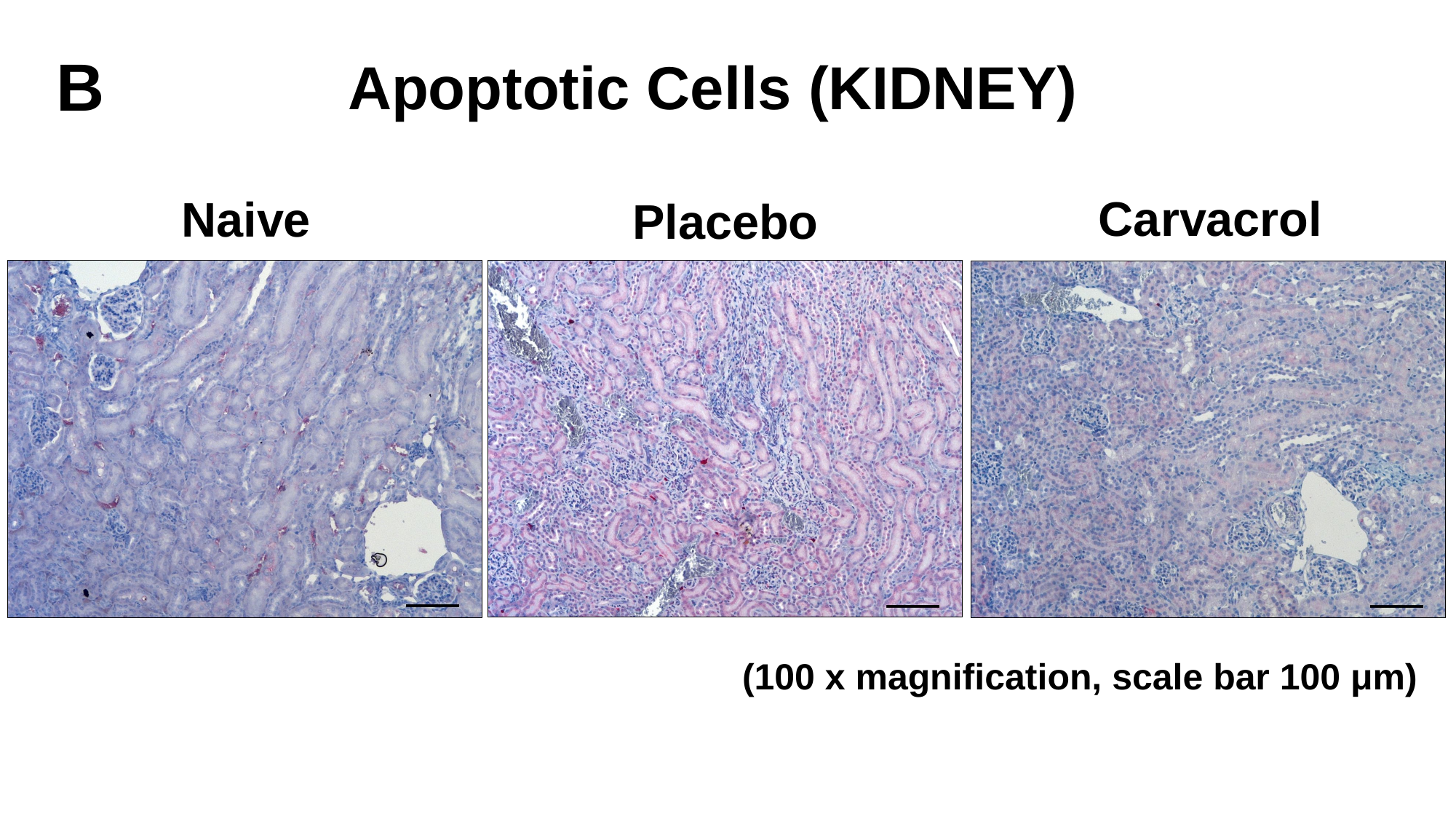

B
Apoptotic Cells (KIDNEY)
Carvacrol
Naive
Placebo
(100 x magnification, scale bar 100 μm)

## Slide 3
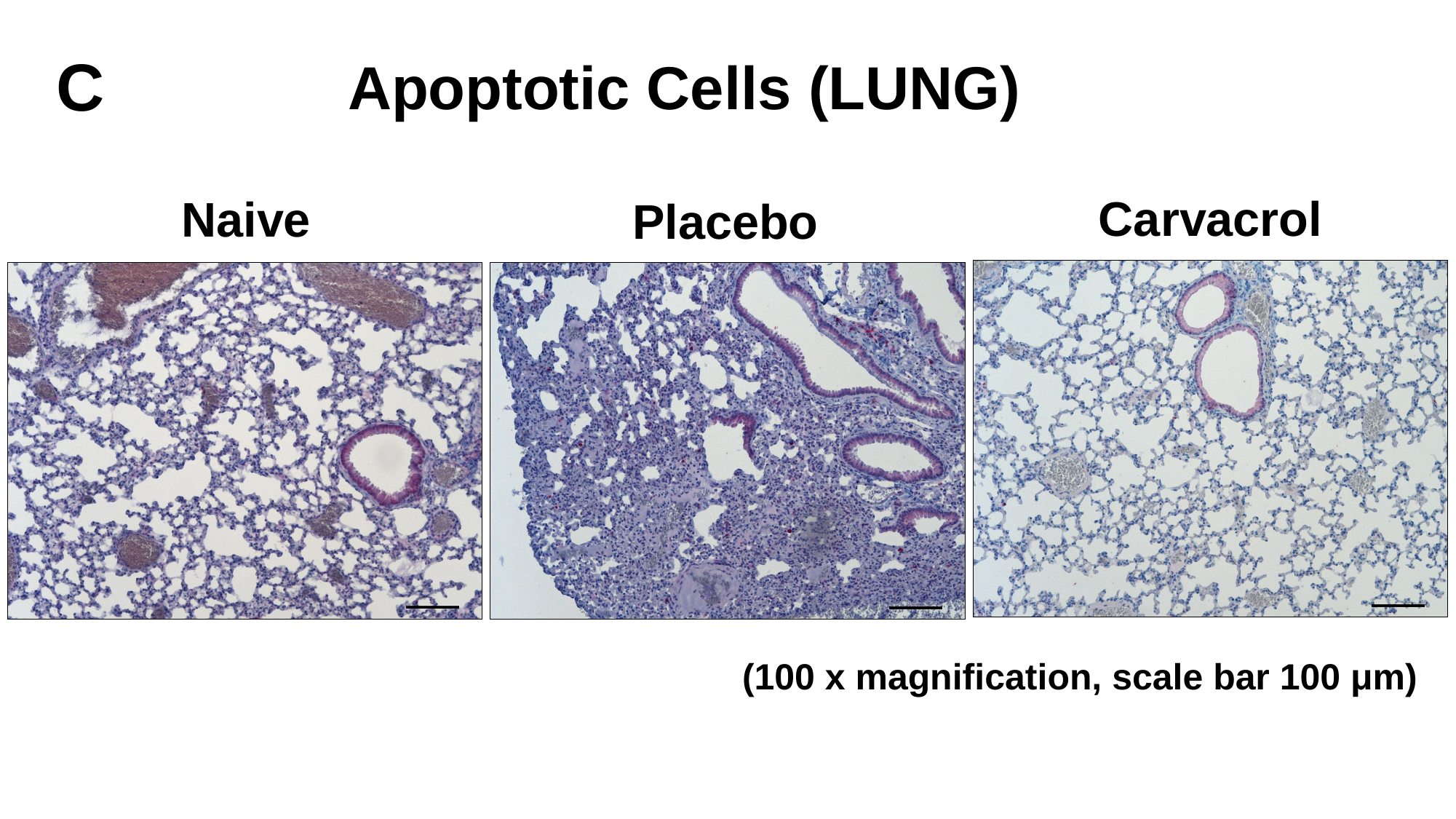

C
Apoptotic Cells (LUNG)
Carvacrol
Naive
Placebo
(100 x magnification, scale bar 100 μm)
